# Supplementary material for: Evaluation of a patient and public involvement training programme for researchers at a large biomedical research centre in the UK
Source: BMJ Open. 2021 Aug 12;11(8):e047995. doi: 10.1136/bmjopen-2020-047995 (PMC8362711; doi:10.1136/bmjopen-2020-047995)
Supplement: Supplementary data [file bmjopen-2020-047995supp001.pdf]

Survey measures

Survey 1 (pre workshop)

1) **Are you currently a Principal/Chief Investigator?**
   Yes
   No
2) **What is your professional role (tick one)?**

   Medical doctor/consultant
   Nurse
   Scientist
   Administrator/study manager
   Statistician/data manager
   Graduate student
   Other (please specify)

3) **How would you describe your attitude towards Patient and Public Involvement (PPI)?**

   PPI enthusiast
   Interested in PPI but have not yet done it
   Indifferent
   Negative

4) **Do you feel able to: (please tick one or more)**

   Confidently carry out PPI
   Work effectively with patients, carers and public$_2$
   Run effective meetings with patients, carers and public
   Listen to patients, carers and public effectively
   Use patients, carers, or the public in steering groups effectively
   Use tools to keep patients, carers and public interested
   Provide accessible information
   None of the above
   Other – please explain:

5) **Do you believe you have a good understanding of what is good practice in PPI?**
   Yes
   No
   A little

6) Do you know what resources are available to help you with PPI?
Yes
No
A little

7) Have you already actively involved patients, carers, or the public in your research?

Yes – go to a)
No

a) What areas did patients, carers, or the public help you with? (please tick one or more)

Identifying topics for research
Prioritising research topics and aims
Designing a research study and protocol
Looking into the ethics of the proposed research and methods
Writing patient information and consent forms
Recruiting participants
Writing grant and research proposals
Having patients, carers, or public as co-applicants
Disseminating results
Other - please explain:

Survey 2 (immediately after workshop)

1) How did you find the workshop? (Please mark the appropriate box for each row)

	Excellent	Good	Satisfactory	Poor
a. Overall workshop	☐	☐	☐	☐
b. Handouts	☐	☐	☐	☐
c. Delivery and presentation of workshop	☐	☐	☐	☐
d. Meeting room and facilities	☐	☐	☐	☐
e. Administration of workshop	☐	☐	☐	☐

2) How would you describe your attitude towards Patient and Public Involvement (PPI) now you have been to this workshop?

PPI enthusiast
Interested in PPI but have not yet done it
Indifferent
Negative

a) Has the workshop changed your attitude? (If yes, please explain)
Yes
No

3) Do you plan to involve patients, carers and the public in your research in the future?

Yes - go to a) and b)
No – go to c)

a) Will this workshop help you to action your plans?
Yes
No

b) How do you think this workshop will help you? (tick one or more)

I have more of an idea about how to involve patients, carers and public
It has given me more confidence to work with patients, carers and public
It has given me practical tips and ideas
I feel better equipped to work with patients, carers and public4
Other – please explain:

4) What abilities and skills did you learn from our workshops? (please tick one or more)

Having confidence to carry out PPI
Understanding how to work with patients, carers and public
Running effective meetings with patients, carers and public
Listening to patients, carers and public more effectively
Using patients, carers and public in steering groups more effectively
Gaining tools to keep patients, carers and public interested
Providing accessible information
None of the above
Other – please explain

5) Would you recommend this workshop to other researchers?
Yes
No

6) **Now that you have attended the workshop**

   a) **Do you believe you have a good understanding of what is good practice in PPI?**
      Yes
      No
      A little

   b) **Do you know what resources are available to help you with PPI?**
      Yes
      No
      A little

7) **Are you currently a Principal/Chief Investigator**
   Yes
   No
8) **What is your professional role (tick one)?**

   Medical doctor/consultant
   Nurse
   Scientist
   Administrator/study manager
   Statistician/data manager
   Graduate student
   Other (please specify): ___________________

9) **Do you have any other comments or suggestions?**

<u>Survey 3 (6 months after workshop)</u>

1. How would you describe your attitude towards Patient and Public Involvement (PPI) since attending the workshop? (Tick one box)

PPI enthusiast
Interested in PPI but have not yet done it
Indifferent
Negative

2. Since you attended the workshop in <month> have you actively involved patients or the public in your research? (Tick one box)

Yes – go to question 3 -
No, I haven't conducted any research since the workshop - go straight to question 7
No, PPI would not help my research - go straight to question 7
No, I don't feel able to actively involve the public - go straight to question 7
No, I didn't for other reasons - please specify and then go to question 7

3. What areas did patients, carers or the public help you with? (Tick relevant boxes)

Identifying topics for research
Prioritising research topics and aims
Designing a research study and protocol
Looking into the ethics of the proposed research and methods
Writing patient information and consent forms
Recruiting participants
Writing grant and research proposals
Having patients, carers, or public as co-applicants
Disseminating results
Other (please specify)

4**. Do you feel the workshop you attended in < month> helped you to involve patients, carers and the public in this way?**

Yes
No

5. How did this PPI influence your research? (Tick relevant boxes)

Patient information/consent forms were re-written
We changed or adjusted study design protocol
We changed or adjusted outcome measures
Patients/public assured us as to the ethical nature of our research and its acceptability
Patients/public gave us access to particular groups of patients and potential participants
We changed our proposed recruitment procedures
Lay people helped recruit participants

Our department/unit made changes to research strategic direction and priorities
PPI had no influence on our research
PPI influenced our research in another way (please specify)

6. What do you anticipate will be the overall impact of this PPI will have on your research? (Tick relevant boxes)

Better recruitment
Better retention
More meaningful outputs with research results more likely to make a difference
Priorities of our department/unit are more likely to be relevant to the needs of patients
Greater credibility with funders and stakeholders
Improved dissemination of research results
Information about research is more accessible
Funding grant secured
More rigorous research
No overall impact
PPI had a negative impact on our research

7. Which aspects of PPI do you now feel able to do and which would you like more training in?

	Able to do this	I need more training
Confidently carry out PPI	☐	☐
Work effectively with patients, carers and public	☐	☐
Run effective meetings with	☐	☐

patients, carers and public		
Listen to patients, carers and public effectively	☐	☐
Use patients, carers, or the public in steering groups effectively	☐	☐
Use tools to keep patients, carers and public interested	☐	☐
Provide accessible information	☐	☐
None of the Above	☐	☐
Other	☐	☐

8. Would you be interested in undertaking further training in PPI? (Tick one box)

Yes
No

9. If you would be interested in more training, what kind of training would you like? (Tick relevant boxes)

More introductory sessions on PPI
More advanced training
More practical training
Training where I can look at and discuss my own PPI plans
Training conducted by patients or members of the public
Sessions where I can exchange ideas with other researchers
Other (please specify)

10. Would you recommend the PPI workshop you attended to other researchers?

Yes
No
