# Supplementary material for: Evaluation of a patient and public involvement training programme for researchers at a large biomedical research centre in the UK
Source: BMJ Open. 2021 Aug 12;11(8):e047995. doi: 10.1136/bmjopen-2020-047995 (PMC8362711; doi:10.1136/bmjopen-2020-047995)
Supplement: Author's manuscript [file bmjopen-2020-047995.draft_revisions.pdf]

BMJ Open is committed to open peer review. As part of this commitment we make the peer review history of every article we publish publicly available.

When an article is published we post the peer reviewers' comments and the authors' responses online. We also post the versions of the paper that were used during peer review. These are the versions that the peer review comments apply to.

The versions of the paper that follow are the versions that were submitted during the peer review process. They are not the versions of record or the final published versions. They should not be cited or distributed as the published version of this manuscript.

BMJ Open is an open access journal and the full, final, typeset and author-corrected version of record of the manuscript is available on our site with no access controls, subscription charges or pay-per-view fees (http://bmjopen.bmj.com).

If you have any questions on BMJ Open's open peer review process please email info.bmjopen@bmj.com

BMJ Open

Impact of training in patient and public involvement on biomedical researchers and their research: findings of evaluation of 4 years of training provision across a major university and NHS trust

Journal:	*BMJ Open*
Manuscript ID	bmjopen-2020-047995
Article Type:	Original research
Date Submitted by the Author:	24-Dec-2020

Complete List of Authors:	Yu, Rosamund; University College London Hospitals NHS Foundation Trust, NIHR biomedical research centre at UCLH<br>McNally, Nicholas; University College London<br>Hanley , Bec ; Medical Research Council Clinical Trials Unit at University College London<br>Denegri, Simon; Academy of Medical Sciences<br>Ahmed, Jaber; University College London Hospitals NHS Foundation Trust

Keywords:	EDUCATION & TRAINING (see Medical Education & Training), Clinical governance < HEALTH SERVICES ADMINISTRATION & MANAGEMENT, Organisational development < HEALTH SERVICES ADMINISTRATION & MANAGEMENT, MEDICAL EDUCATION & TRAINING, STATISTICS & RESEARCH METHODS

SCHOLARONE™
Manuscripts

BMJ

I, the Submitting Author has the right to grant and does grant on behalf of all authors of the Work (as defined in the below author licence), an exclusive licence and/or a non-exclusive licence for contributions from authors who are: i) UK Crown employees; ii) where BMJ has agreed a CC-BY licence shall apply, and/or iii) in accordance with the terms applicable for US Federal Government officers or employees acting as part of their official duties; on a worldwide, perpetual, irrevocable, royalty-free basis to BMJ Publishing Group Ltd ("BMJ") its licensees and where the relevant Journal is co-owned by BMJ to the co-owners of the Journal, to publish the Work in this journal and any other BMJ products and to exploit all rights, as set out in our licence.

The Submitting Author accepts and understands that any supply made under these terms is made by BMJ to the Submitting Author unless you are acting as an employee on behalf of your employer or a postgraduate student of an affiliated institution which is paying any applicable article publishing charge ("APC") for Open Access articles. Where the Submitting Author wishes to make the Work available on an Open Access basis (and intends to pay the relevant APC), the terms of reuse of such Open Access shall be governed by a Creative Commons licence – details of these licences and which Creative Commons licence will apply to this Work are set out in our licence referred to above.

Other than as permitted in any relevant BMJ Author's Self Archiving Policies, I confirm this Work has not been accepted for publication elsewhere, is not being considered for publication elsewhere and does not duplicate material already published. I confirm all authors consent to publication of this Work and authorise the granting of this licence.

For peer review only

Title: Impact of training in patient and public involvement on biomedical researchers and their research: findings of evaluation of 4 years of training provision across a major university and NHS trust

Authors
Rosamund M Yu (corresponding and lead author and guarantor)
Rosamund.yu@ucl.ac.uk
Head of PPI and Research Communications, National INstitue for Health Research biomedical research centre at University College London Hospitals NHS Foundation Trust
1st Floor, Suite A, Maple House, 149 Tottenham Court Road, London W1T 7DN
Tel: 07962943966
Fax: 020 7380 9937

Nicholas J McNally
Managing Director, Research UCLH/UCL
National Institue for Health Research Biomedical Research Centre at University College London Hospitals NHS Foudnation Trust
1st Floor, Suite A, Maple House, 149 Tottenham Court Road, London W1T 7DN, UK

Bec Hanley
freelance adviser on patient involvement
National Institue for Health Research Biomedical Research Centre at University College London Hospitals NHS Foudnation Trust
1st Floor, Suite A, Maple House, 149 Tottenham Court Road, London W1T 7DN, UK

Simon Denegri
Executive Director, Academy of Medical Sciences
41 Portland Pl
London W1B 1Q, UK

Jaber Ahmed
Digital Communications Officer National Institue for Health Research Biomedical Research Centre at University College London Hospitals NHS Foudnation Trust
1st Floor, Suite A, Maple House, 149 Tottenham Court Road, London W1T 7DN
Contribution: Author carried out analysis of statistical findings.

Special thanks to patient advisors Libby Cooper, Brenden Conroy, Philip Creasy, Anthony Locke,

Key words
Patient Involvement
Research personnel
Biomedical research
Continuing medical education

Word count – (excluding title page, references, figures tables, contributorship, competing interest and data statements): 4109

For peer review only - http://bmjopen.bmj.com/site/about/guidelines.xhtml

Abstract

Objectives A programme of training for researchers on actively involving patients and the public (PPI) was established with the aim of developing high quality effective PPI that would improve biomedical research at a large NHS trust and partner university.
Design Workshops were designed to build practical skills and confidence to work with patients and the public. **Outcome measures** Surveys before, immediately after and 6 months after training looked at the impact on researchers' confidence and skills in PPI. Surveys six months after also looked at PPI carried out, and its impact on research.
Setting Workshops took place across UCL, UCLH and UCL partner organisations.
Participants Between 2014 and 2018, 72 workshops were held, training 721 scientists, clinicians and research managers.
Results Training brought about high increases (in excess of 400%) in researchers' confidence to carry out PPI, and knowledge of good practice.
6 months after training, over 60% of respondents had involved the public in their research and levels of confidence and skills with 2 exceptions were higher than immediately after training. Over 60% of respondents involved patients in looking at research priorities but, only 5-20% said their department had made changes to research strategy and priorities. Some 29%-36% had involved people in writing patient information and over 30% had rewritten patient information.

Conclusions Practical PPI training can have greatest impact on researchers' confidence in involving patients in their work. It is a key enabler for researchers to experience first-hand the value of PPI to research and can directly impact on research.
Training, together with direct experience of PPI, can lead to a braver approach, with a high proportion of researchers taking on the challenge of PPI in research strategy setting. However, this had a limited impact on research, compared with PPI in patient information and study design.

Strengths and Limitations

- This study evaluates probably one of the largest PPI in research training programmes in the UK across multiple institutions, and looked at feedback from over 700 researchers on the impact of training.
- Evaluation was carefully designed to monitor the impact of PPI training on researchers and their work.
- Response rates to the before and after survey were high (average 98%) – but for the 3rd optional survey 6 months after training the response rate was much lower (average 36%) and respondees were self-selecting.
- Evaluation depended on researchers' self-evaluation.
- Because this work was not intended to be research, slight differences in question wording may have caused anomalies.

Introduction

In 2014 the National Institute for Health Research University College London Hospitals Biomedical Research Centre (BRC) started running a rigorously monitored training programme for researchers on actively involving patients and the public in research (PPI). Nationally, at that time, PPI was increasingly encouraged and promoted but was not yet considered the 'norm' in research, despite reports such as The NHS Constitution and the Francis report encouraging a greater focus on engaging with patients, carers and the public[1] in research. The principal arguments for PPI in

For peer review only

research were, and in fact still are, that it improves the quality and effectiveness of health research, ensuring the relevance of research to the needs of patients, carers and the public, with a greater likelihood of research findings being put into practice, so improving health and wellbeing[2]. Director General of Research and Development, Chief Scientific Advisor for the Department of Health and NHS Dame Sally Davis, asserted in 2009 that "No matter how complicated the research, or how brilliant the researcher, patients and the public always offer unique, invaluable insights. Their advice when designing, implementing and evaluating research invariably makes studies more effective, more credible and often more cost effective.[3]"

One of the factors holding back PPI in clinical research has undoubtedly been a lack of understanding of PPI among researchers. A 2011 study by the National Research Ethics Service and national advisory group INVOLVE revealed that while 19% of researchers were shown to be actively involving patients, another 43% said they were involving patients and the public but were mistaking engagement and recruitment activities for active involvement. [4]. The need for dedicated PPI training for researchers has long been identified as a priority for clinical research in the UK [5] and the Concordat for Engaging the Public with Research, to which Research Councils UK is a signatory[6], strongly advocated it. The value of training in PPI has also been demonstrated internationally, for example in Australia where one study found training increased awareness and supportive attitudes for involvement[7]. But researchers themselves did not always view training as necessary. Dudley et al for example found a considerable portion of researchers with no training in involvement did not want training, believing they already knew how to 'do' PPI[8].

The research charity sector in the UK has in many respects led the way in promoting patient involvement in research, with comprehensive, targeted support and training for researchers and public contributors. However, the BRCs operate in a very different environment. While medical research charities are focused on single diseases, a large BRC like that at UCLH covers a wide range of clinical specialties, disease areas and disease sub-types. At any one time, UCLH has 1,200 studies open, opening 300 new studies and recruiting 15,000 patients each year. This magnitude and complexity, while offering significant opportunities for PPI in research, meant the BRC initially faced logistical challenges in terms of knowing what PPI was happening and in attracting researchers into training. The UCLH BRC identified patient and public involvement as a key part of its strategy to speed up the translation of science into better patient care and to ensure that its research addressed patients' healthcare needs. PPI training for researchers was identified as a way of developing the research infrastructure.

Early interactions with research groups revealed a lack of confidence about PPI. Researchers cited uncertainty about finding people to involve in studies and how to work with them. PPI was often only considered hurriedly ahead of submission deadlines for research grant applications. Against this back drop the UCLH BRC developed bespoke training as a way of empowering researchers to conduct high quality PPI.

Methods

The BRC's strategy of developing a programme of PPI training workshops, rather than ad hoc events, was a direct result of the BRC's strategy to take an imaginative, long term approach to the challenge of developing capability and capacity for PPI in the research workforce.

The programme of workshops was delivered for the first 3 years by two PPI training experts in the light of results of a 2012 training needs survey of 100 UCL and UCLH health researchers and work with patients who had already worked with researchers. Workshops were designed to be short, and to include input from a lay person and a researcher. Workshops were targeted with an emphasis on trying to 'get people through the door' and focused on practical tools for PPI rather

For peer review only - http://bmjopen.bmj.com/site/about/guidelines.xhtml

than on its philosophical underpinnings. Trainers took a flexible approach, continually adapting workshops to the needs of researchers.

The BRC's training programme was large-scale and run over each academic term at multiple sites, reflecting a growing interest in PPI. Since 2014 a total of 72 workshops, training 721 scientists, clinicians and research managers have been delivered. The programme won an award from Health Education North Central and East London for its work in "education and training that has been innovative in putting patients and/or carers at the centre of the programme," and gained institutional recognition in 2018, with UCL awarding a career point to researchers for every half day workshop attended.

Evaluation was embedded in the design of the programme and aimed to find out whether the workshops ultimately enabled researchers to carry out good quality, effective focusing on:

- Did training build up confidence and knowledge and enable researchers to carry out PPI they could not have done before?
- What kind of PPI did they carry out and what effect did it have on research?

On arrival, each participant was handed a survey to complete before the workshop (survey 1) and a sealed envelope containing a survey to fill in after the workshop (survey 2). A response rate of on average 98% was achieved for surveys 1 and 2. Six months after the workshops, a 3rd survey (survey 3) was sent to all participants using Survey Monkey and a response rate of on average 36% was achieved. Telephone interviews were carried out for more in-depth feedback.

Patient and Public Involvement

Training was developed and carried out in partnership with patients.

- The training was designed after working with patients. Surveys and focus groups of lay people who had previously worked with researchers highlighted skills and areas patients felt researchers needed to be trained in. This input shaped not only the nature of the training delivered but also what was measured in terms of impact. A good example is researchers' communication skills, which patients had prioritised.
- Sessions were formatted so that the 2 trainers, who included a patient advocate, delivered training in partnership with a patient.

Results

Workshops were initially delivered over a 1-2 month period while in subsequent years, the programme was designed to cover each academic year. Consequently, survey results have been analysed by calendar years 2014 and 2015 and by academic years, 2016/17 (May 2016-June 2017) and 2017/18 (September 2017- May 2018). Full results from Survey 3 were not available for 2017/18.

Detailed analysis of first year of training (2014)

Workshops attracted a range of professionals from the clinical and academic workforce at UCLH/UCL and partner NHS trusts and universities, including Moorfields Eye Hospital, Great Ormond Street Hospital for Children and Queen Mary University of London.

For peer review only - http://bmjopen.bmj.com/site/about/guidelines.xhtml

- Over 40% of attendees did not fit into the usual scientist/clinician/administrator groups. Many were allied health professionals. Answers included but were not limited to: Dietician, Dentist, Public Health Specialist, Physiotherapist, Therapy Manager, Rehabilitation Assistant, Recruitment officer and Psychologist.

- 18% were scientists and 20% described themselves as a clinician.

Over 90% of participants rated workshops very good or excellent.

1. Immediate impact on attitudes and skills

Both the first and the second surveys asked about attitudes, understanding and competencies in PPI, to see whether training brought about any changes.

Although attitudes to PPI remained largely stable, understanding and confidence increased after training (figure 1).

- 20% of people arriving at workshops felt they had a good understanding of PPI, rising to 90% immediately after the workshop

- before training 9% of people felt they understood the resources available to support PPI, rising to 71% immediately after training.

The section on PPI skills revealed more detail (figure 2). Participants arriving at the workshop were asked to select from a list the skills they thought they had in PPI. On leaving they were asked what skills they had learnt in the workshop.

Areas where there was a change before and after the workshop were in confidently carrying out PPI (up from 10% to 39%), using tools to keep patients interested (from 16% to 33%); and running effective meetings with patients (from 20% to 32%).

There was a drop after training in researchers' assessment of two skill areas – their ability to listen to patients (down from 54% to 30%) and their ability to provide accessible information (down from 44% to 28%).

98% of participants planned to involve patients, and the public in their research.

2. Six months later – what was the long-term impact?

Results included:

I. PPI activities (figures 3 and 4)

Since the workshop 64% of researchers responding to the survey had carried out PPI. 20% had not carried out PPI because they had not done any research and one researcher had not carried out PPI because they felt unable to.

The main areas researchers had involved patients in were: identifying and prioritizing research topic and aims, and designing a study protocol.

Only 29% had involved people in writing patient information and consent forms with 30% re-writing patient information. 37% had involved people in the design of a

For peer review only - http://bmjopen.bmj.com/site/about/guidelines.xhtml

study and protocols, and 38% of respondents had changed or adjusted their study design.

47% of respondents had involved patients and the public in identifying research topics and 39% had involved them in prioritising research topics and aims. Only 5% of respondents said their department or unit had made changes to research strategic direction and priorities. However, PPI had provided many with reassurance that their research was acceptable and ethical (27%).

ii. predicted impact (figure 5)

63% of respondents anticipated the PPI would ensure more meaningful outputs more likely to make a difference. 45% predicted their research would have greater credibility with funders and stakeholders. Less (11%) felt it would actually help them secure funding. A third envisaged better dissemination and more accessible information.

iii. Changes to skills and confidence 6 months after training

Confidence to carry out PPI, was higher 6 months after training, rising from 39% to 64% in that period. Researchers were most confident about their ability to listen to patients (over 75%).

Trends in survey results for subsequent years (2014-2018)

Between 2015 and 2018 evaluation results were comparable to those of 2014 but figures 1-5 reveal changes.

Results demonstrate training continued to build researchers' confidence and suggest a growing impact of PPI on trial recruitment and retention and departmental strategic priorities.

The high increase in the proportion of participants who felt they had an understanding of PPI after training observed in 2014 results continued, as did the increase in the proportion of researchers who felt that they could confidently carry out PPI after attending a workshop with the proportion increasing from 11% before the workshop to 70% afterwards in 2016/17.

Base line confidence before training in some skill areas varied over the 3 periods. Whatever the baseline, workshops still increased researchers' confidence levels especially in working effectively with patients and running effective meetings, where confidence could sometimes double or go up by a third.

Another pattern repeated in 2015 and 2016/17 is the dent immediately after training to researchers' confidence in their ability to listen to patients and carers and to provide accessible information, followed by an increase 6 months later.

From 2014-2016/17 the proportion of participants confident in their ability to listen to patients, carers and the public tended to fall by about a third after training Consistently, the proportion confident in this area increased 6 months after the workshop, rising from between 30% and 45% immediately after training to between 52% and 90% 6 months after training. For providing accessible information levels rose to between 50 and 79%.

For peer review only - http://bmjopen.bmj.com/site/about/guidelines.xhtml

The percentage of participants carrying out PPI in the 6 months after training always exceeded 60%. What did change was the percentage who said they did not carry out PPI because it would not help their research falling to 3% in 2016/17.

The sphere of influence PPI had on research and the nature of that impact remained consistent with some adjustments over the 3 periods. The main areas patients helped researchers with continued to be: prioritising research topics and aims; designing a study and protocols; and writing patient information and consent forms. There was a fall in the proportion of researchers involving the public in identifying topics for research (47% in 2014 to 24% in 2016/17). However, there was a rise (39% in 2014 to 47% in 2016/17) in the proportion involving the public in prioritising research topics.

The main impacts of PPI continued to be: patient information or consent forms re-written (between 30 and 35%); study design and protocol changed (30-41%); assurance by patients as to acceptability of research (24-29%); and access to particular patient groups (15-30%).

For anticipated overall impact, over 60% said PPI would cause more meaningful outputs with research more likely to make a difference. 45-52% said PPI would mean their research would have greater credibility with funders and stakeholders and 29-50% said it would lead to better recruitment. 29-44% said PPI would mean the priorities of their department were more likely to be relevant to the needs of patients.

Changes over the 3 periods to the long term impact envisaged included notable increases in the proportion anticipating better recruitment (from 29% to 50%) better retention (from 16 % to 30%), department priorities more likely to be relevant to the needs of patients (from 30 to 44%),and securing of funding (from 11% to 19%). There was a slight decrease in the proportion saying PPI would lead to more accessible information about research.

Discussion

For peer review only - http://bmjopen.bmj.com/site/about/guidelines.xhtml

Key findings:

- Before and immediately after training over the 3 periods analysed:
  - High increases (in excess of 400%) in confidence to carry out PPI, understanding of good practice and knowledge of resources
  - Increases in ability to work effectively with patients (to over 50%), using patients in steering groups (up to 33% or more), using tools to keep patients interested (up to 33% or more)
  - Fall in ability to listen to patients, carers and the public effectively and provide accessible information
- Six months after training, over the 3 periods:
  - Over 60% had involved patients, carers and the public in their research
  - Levels of confidence and skills with 2 exceptions were higher than immediately after training, including (except in 2016/17) ability to listen to patients, carers and the public effectively and provide accessible information, which had dropped after training.
  - Researchers involved the public in prioritising research topics and aims (between 39% - 51%); designing a study and protocols (37- 60%); and writing patient information (29-51%)
  - PPI impacted on research: patient information or consent forms were re-written (30-35%); study design and protocols changed (30-41%); assurance as to acceptability of research (24-29%); and access to particular groups of patients (15-30%).
  - Over 60% of respondents anticipate their research would have more meaningful outputs with research more likely to make a difference.

Analysis of surveys of researchers attending PPI training at UCL/UCLH demonstrates that this PPI training built up researchers' confidence in actively involving patients in their work.

Training increased researchers' understanding of how to effectively work with patients and the public, and enabled them to develop a range of skills and knowledge.

The fall after training in confidence in 2 skills – ability to listen to patients, carers and the public and ability to provide accessible information - may reflect the wording of the question in the after- training survey which asked what skills they had gained. However, it is also likely that the training stimulated researchers to question their communication skills.

Six months after training, results show that most confidence levels were even higher than immediately after training. Moreover the observed dent to confidence in communications skills after training had been reversed with confidence levels higher than before training.

Although it is clear from the results that training built confidence and understanding, the higher levels shown in surveys 6 months after the training when over 60% had carried out PPI suggest that first-hand experience of working with patients and the public was a valuable skills promoter, and that a valuable impact of initial training was to give researchers the confidence to carry out PPI and build up further confidence. A conclusion endorsed by the reversal of the drop in confidence in 2 communication skill areas.

For peer review only - http://bmjopen.bmj.com/site/about/guidelines.xhtml

Further weight is given to these findings in that comparable results were achieved in the following years. Whatever the level of base line confidence, training always increased that level.

Of note is that over the 3 periods consistently over 25% had involved patients in identifying research topics and over 39% had involved patients in prioritizing research topics. If these options are considered mutually exclusive with researchers opting for either one or the other, this would suggest over 60% of respondents involved patients in looking at research priorities in some way. As this is arguably one of the most challenging kinds of PPI, this was an exciting and promising finding. It suggests that confidence building inspires a braver approach to involvement. However, the impact was limited with only 5-20% of respondents reporting that their department or unit had made changes to research strategic direction and priorities. Over the 3 periods, researchers moved away from involving the public in identifying research topics to involving the public in prioritizing research topics, suggesting a more realistic approach to what could be achieved.

A smaller proportion (29%-36%) had involved people in writing patient information and consent forms – a traditional area of PPI, often where researchers tend to start and where there is something of a guaranteed impact. Over 30% had rewritten patient information. A similar guaranteed impact was observed in patient input into design of a research study and protocols.

The increase over the 3 periods to final levels of confidence 6 months after training also probably reflects a growing cohort of researchers increasingly adept at involving patients and the public. This confidence, the authors would suggest, leads not only to a broader, braver approach to involving patients and the public, but also to an optimism for the future impact of PPI. Increasingly, researchers anticipated a greater impact on the very tangible area of recruitment and retention; the relevance of departmental research priorities to patient need; and securing of funding.

One limitation of findings from the surveys used is that they were subjective evaluations by the researchers themselves. How impact can be measured without subjective evaluation remains a methodological challenge. The other limitation is that the first and second surveys, before and immediately after training, achieved a high response rate, while this was much lower for the survey 6 months after training. Comparisons have been made between the surveys, although confidence in this must be limited because of the lower response rate and the fact that those responding to the 3rd survey were self-selecting, in that they chose to respond to an email and were probably more interested in PPI than non-responders.

The BRC's experience of developing training for researchers in PPI demonstrates that within a context of resistance and nervousness about PPI it is possible to achieve effective capacity building of PPI skills among biomedical researchers, even within large and complex institutional structures. Moreover, it is also possible to gain institutional recognition of training as a fundamental part of researchers' continuing professional development. Developing the capacity to involve patients can and does have an impact on research, in particular leading to changes to study design and patient information, prioritising of research topics and help with recruitment.

As yet there is no evidence available to demonstrate how beneficial to research these impacts are, or indeed whether patients perceived any impact on research. These are both areas the authors would recommend for further evaluation. Many research institutions are

For peer review only - http://bmjopen.bmj.com/site/about/guidelines.xhtml

starting to provide the evidence for how PPI can make research better. This article demonstrates that in order to get researchers involving patients and to ensure that PPI has an impact on research, it is essential to build up confidence, practical knowledge and the capacity to experience first hand the value of PPI to research. This pragmatic approach can only lay the foundations for the future by equipping a generation of researchers to be able and willing to involve patients and the public in their work.

A key recommendation is that training of researchers should be considered a fundamental way of developing the involvement of the public in research. Long term strategic thinking and investment should be the basis for the establishment of training. The authors would strongly recommend that, to be effective, training should be tailored to give researchers practical tools rather than to lecture on the moral and ethical imperative of PPI.

Contributorship statement
Rosamund M Yu (corresponding and lead author and guarantor) lead contribution: established and managed the training programme, designed the evaluation of the programme and carried out the analysis of findings, and wrote the first and final drafts of the article.

Nicholas J McNally equal contribution: led and oversaw the directorate carrying out the training programme and managed its final outcome, and steered and edited the article from draft to final stage

Simon Denegri equal contribution: leader of the work area and advised on production of the training, and its analysis, and edited and steered the article from draft to final stage.

Bec Hanley equal contribution: the trainer commissioned, with another trainer, to design and carry out the training, including its evaluation, and and edited and steered the article from draft to final stage.

Jaber Ahmed equal contribution: carried out analysis of statistical findings.

Special thanks to patient advisors Libby Cooper, Brenden Conroy, Philip Creasy, Anthony Locke.

Competing interests
All authors have completed the Unified Competing Interest form and, with 2 exceptions, declare: no support from any organisation for the submitted work; no financial relationships with any organisations that might have an interest in the submitted work in the previous three years], no other relationships or activities that could appear to have influenced the submitted work]. One author discloses that they were a paid employee of the institution running the training discussed at the time this work was undertaken. Another author discloses that they received a fee to deliver the training discussed in the article.
Ethical approval and study sponsors - NA

Funding
National Institue for Health Research biomedical research centre at University College London Hospitals NHS Foundation Trust
Health Education North Central and East London

For peer review only - http://bmjopen.bmj.com/site/about/guidelines.xhtml

Wellcome grant number 204841/Z/16/Z.

Data Sharing Statement
Data is made up of answers to surveys by researchers attending training. Attendees are not identifiable. Data is not in a repository and can be requested from Rosamund.yu@ucl.ac.uk

Figure captions
Figure 1 Knowledge and understanding of PPI good practice and resources
Figure 2 PPI skills
Figure 3 Kinds of PPI carried out
Figure 4 Impact of PPI on research
Figure 5 Anticipated future impact of PPI on research

---

[1] Health and Social Care Act 2001, the NHS Constitution for England and National Health Service Act 2006, including Real Involvement – Working with People to Improve Health Services October 2008 (Guidance for NHS organisations on section 242(1B) of the NHS Act 2006, the duty to involve and good involvement practice), The Francis Report - Report of the Mid Staffordshire NHS Foundation Trust Public Inquiry, February 2013
[2] HRA Strategy for Public Involvement, version 1.1, Health Research Authority, 2013
[3] Staley K. Exploring impact: public involvement in NHS, public health and social care research. Eastleigh: INVOLVE, 2009
[4] Tarpey M., (2011) Public involvement in research applications to the National Research Ethics Service, INVOLVE, Eastleigh

[5] . INVOLVE survey of priorities for public involvement across the NIHR September 2011
[6] Concordat for Engaging the Public with Research
[7] McKenzie, A., Alpers, K., Heyworth, J. *et al.* Consumer and community involvement in health and medical research: evaluation by online survey of Australian training workshops for researchers. *Res Involv Engagem* **2,** 16 (2016) doi:10.1186/s40900-016-0030-2

[8] Dudley, L., Gamble, C., Allam, A. *et al.* A little more conversation please? Qualitative study of researchers' and patients' interview accounts of training for patient and public involvement in clinical trials. *Trials* **16,** 190 (2015) doi:10.1186/s13063-015-0667-4

For peer review only - http://bmjopen.bmj.com/site/about/guidelines.xhtml

Figure 1

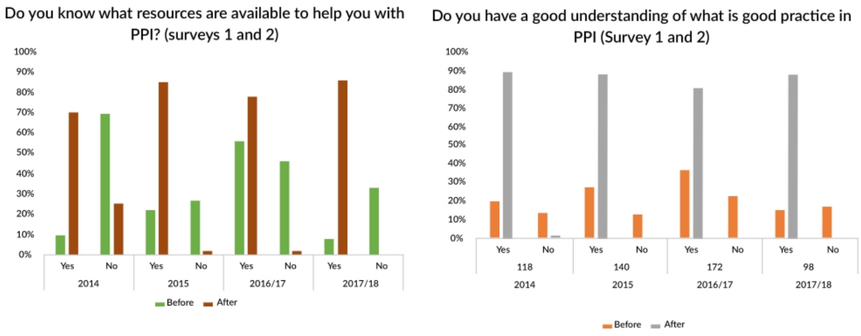

Figure 1Knowledge and understanding of PPI good practice and resources

297x147mm (300 x 300 DPI)

Figure 2

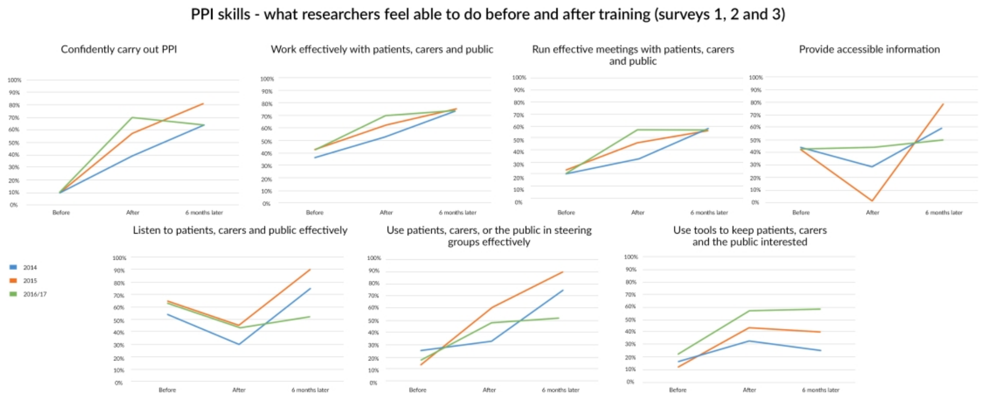

Figure 2 PPI Skills

297x147mm (300 x 300 DPI)

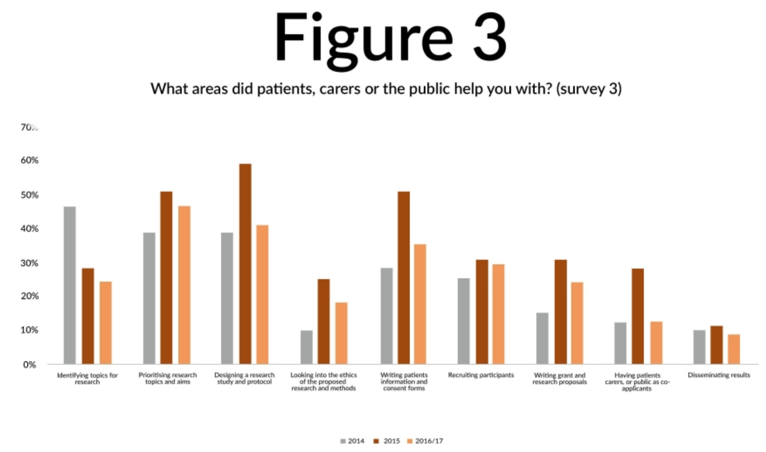

Figure 3 Kinds of PPI carried out

297x147mm (300 x 300 DPI)

Figure 4

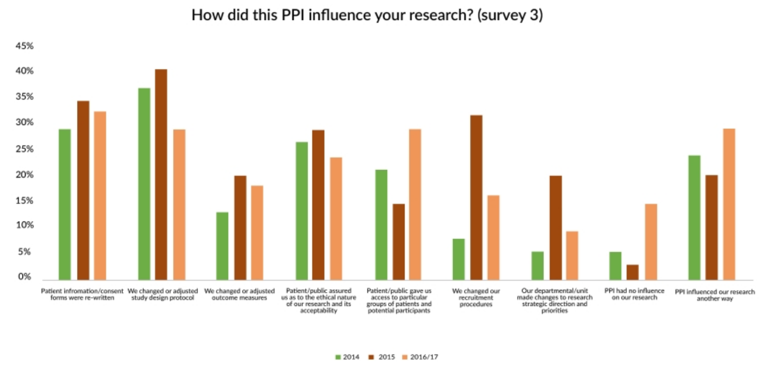

Figure 4 Impact of PPI on research

297x147mm (300 x 300 DPI)

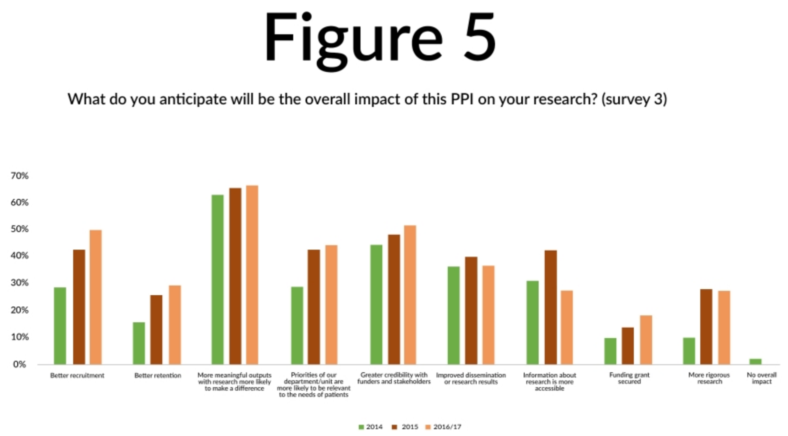

Figure 5 Anticipated future impact of PPI on research

297x147mm (300 x 300 DPI)

The impact of patient and public involvement training on biomedical researchers: findings of an evaluation of 4 years of training

Journal:	*BMJ Open*
Manuscript ID	bmjopen-2020-047995.R1
Article Type:	Original research
Date Submitted by the Author:	26-Apr-2021
Complete List of Authors:	Yu, Rosamund; University College London Hospitals NHS Foundation Trust, NIHR biomedical research centre at UCLH<br>Hanley , Bec ; Medical Research Council Clinical Trials Unit at University College London<br>Denegri, Simon; Academy of Medical Sciences<br>Ahmed, Jaber; University College London Hospitals NHS Foundation Trust<br>McNally, Nicholas; University College London
**Primary Subject Heading**:	Research methods
Secondary Subject Heading:	Patient-centred medicine, Medical education and training
Keywords:	EDUCATION & TRAINING (see Medical Education & Training), Clinical governance < HEALTH SERVICES ADMINISTRATION & MANAGEMENT, Organisational development < HEALTH SERVICES ADMINISTRATION & MANAGEMENT, MEDICAL EDUCATION & TRAINING, STATISTICS & RESEARCH METHODS

SCHOLARONE™
Manuscripts

I, the Submitting Author has the right to grant and does grant on behalf of all authors of the Work (as defined in the below author licence), an exclusive licence and/or a non-exclusive licence for contributions from authors who are: i) UK Crown employees; ii) where BMJ has agreed a CC-BY licence shall apply, and/or iii) in accordance with the terms applicable for US Federal Government officers or employees acting as part of their official duties; on a worldwide, perpetual, irrevocable, royalty-free basis to BMJ Publishing Group Ltd ("BMJ") its licensees and where the relevant Journal is co-owned by BMJ to the co-owners of the Journal, to publish the Work in this journal and any other BMJ products and to exploit all rights, as set out in our licence.

The Submitting Author accepts and understands that any supply made under these terms is made by BMJ to the Submitting Author unless you are acting as an employee on behalf of your employer or a postgraduate student of an affiliated institution which is paying any applicable article publishing charge ("APC") for Open Access articles. Where the Submitting Author wishes to make the Work available on an Open Access basis (and intends to pay the relevant APC), the terms of reuse of such Open Access shall be governed by a Creative Commons licence – details of these licences and which Creative Commons licence will apply to this Work are set out in our licence referred to above.

Other than as permitted in any relevant BMJ Author's Self Archiving Policies, I confirm this Work has not been accepted for publication elsewhere, is not being considered for publication elsewhere and does not duplicate material already published. I confirm all authors consent to publication of this Work and authorise the granting of this licence.

For peer review only

Title: The impact of patient and public involvement training on biomedical researchers: findings of an evaluation of 4 years of training

Authors

Rosamund M Yu (corresponding and lead author and guarantor)

Rosamund.yu@ucl.ac.uk
Head of PPI and Research Communications, National Institue for Health Research Biomedical Research Centre at University College London Hospitals NHS Foundation Trust
1st Floor, Suite A, Maple House, 149 Tottenham Court Road, London W1T 7DN
Tel: 07962943966
Fax: 020 7380 9937

Bec Hanley
Freelance adviser on patient involvement
National Institute for Health Research Biomedical Research Centre at University College London Hospitals NHS Foudnation Trust
1st Floor, Suite A, Maple House, 149 Tottenham Court Road, London W1T 7DN, UK

Simon Denegri
Executive Director, Academy of Medical Sciences
41 Portland Place
London W1B 1QH, UK

Jaber Ahmed
Digital Communications Officer National Institue for Health Research Biomedical Research Centre at University College London Hospitals NHS Foudnation Trust
1st Floor, Suite A, Maple House, 149 Tottenham Court Road, London W1T 7DN
Contribution: Author carried out analysis of statistical findings.

Nicholas J McNally
Managing Director, Research UCLH/UCL
National Institute for Health Research Biomedical Research Centre at University College London Hospitals NHS Foudnation Trust
1st Floor, Suite A, Maple House, 149 Tottenham Court Road, London W1T 7DN, UK

Key words

Patient Involvement

Research personnel
Biomedical research
Continuing medical education

Word count – (excluding title page, references, figures tables, contributorship, competing interest and data statements): 3886

For peer review only

Abstract

Objectives: A programme of training workshops for researchers on actively involving patients and the public (PPI) was established with the aim of building confidence and skills amongst biomedical researchers.

Design: A bespoke programme of training workshops in patient and public involvement aimed at researchers.

Setting: A large National Institute for Health Research Biomedical Research Centre in London and several partner organisations.

Participants: 721 scientists, clinicians and research managers attending dedicated training in patient and public involvement at a major London NHS-university partnership.

Interventions: A programme of 72 training workshops, designed to build practical skills and confidence for researchers working with patients and the public in research, was delivered at a major research-active NHS:university partnership. An iterative approach was taken to the programme, with the content of the workshops continually reviewed and refreshed to respond to the needs of researchers. Surveys before, immediately following and 6 months after training investigated the impact on researchers' confidence and skills in PPI work, and the kind of PPI they subsequently carried out.

Results: Training brought about immediate marked increases in researchers' self-reported confidence to carry out PPI activities within their research, and in their knowledge of good practice. The evaluation indicates that workshop attendees were more likely to involve patients in their research following training. Researchers tended to involve patients and the public in a range of areas, including input to study design and patient information, in particular.

Conclusions: When positioned within a broader organisational strategy for PPI in research, such training has an important role to play in progressing PPI in a major research partnership. Training appeared to provide the confidence needed to carry out PPI which enabled further development of confidence and skills. Involving researchers who have been trained in the on-going development of the training programme and bringing in patients to the on-going training programme are key next steps.

Strengths and Limitations

- This study evaluates probably one of the largest PPI in biomedical research training programmes in the UK, carried out across a major research partnership.
- The precise content of the training workshops was iterative and evolved throughout the programme in response to feedback from participants, making it highly tailored to researchers' needs
- The evaluation was carefully designed to monitor the impact of PPI training on researchers and their work and it did not seek the perspectives of patients participating in projects being carried out by the researchers.
- Response rates to the surveys carried out immediately before and after training were high (average 98%).
- Responses to evaluation surveys were dependent on the researchers' self-evaluation and recall and response rates to the evaluation survey carried out at 6 months post-training were low (34%) and respondents were self-selecting.

For peer review only

INTRODUCTION

In the UK, patient and public involvement (PPI) in biomedical research has been encouraged and promoted over the last 15 years. Evidence of PPI has become a condition of many research funders, notably the National Institute for Health Research (NIHR), which has helped to set expectations for PPI [1]. The NIHR's standards for public involvement are designed to improve quality and consistency of PPI in health research, emphasizing the importance of inclusive opportunities, working together, support and learning, communications, impact and governance, as values-based areas for reflection and learning for researchers and research organisations [2]. This article reports on how a large NHS-University partnership in the UK has, through its NIHR Biomedical Research Centre, developed and deployed an extensive training programme in PPI for research staff as a major component embedding a more extensive culture of PPI in research.

There are two dominant narratives underpinning the importance of patient and public involvement in biomedical research. First is the notion that PPI improves quality, relevance and acceptability of research [3,4,5]. The second is that PPI enables citizens to exercise their rights. This can mean that they feel empowered and valued and that they can build their skills and/or knowledge. [6,7]. The power dynamic that exists between healthcare professionals, researchers and patients can be deep rooted [8,9,10]. The primacy that is given to clinical or scientific knowledge over the experiential knowledge that patients bring to the research process has been shown to render much involvement practice as tokenistic [11]. There are considerable challenges of establishing meaningful PPI in hierarchical, scientific research organisational settings [12].

Researchers' experiences and attitudes towards PPI are undoubtedly key to embedding involvement within the wider research culture of a research organisation [13]. Dedicated training in PPI has long been identified as a need [14] and an important mechanism for developing researchers' skills, experiences and attitudes to PPI [15,16,17,18,19]. Researchers are also being challenged to document in a structured way how they involve patients throughout the research process to ensure PPI practice is based on the best evidence [20,21].

In England, the National Institute for Health Research Biomedical Research Centres (BRCs) are partnerships between NHS Trusts and universities. The BRCs are funded by the NIHR to drive experimental medicine research, taking promising scientific concepts from laboratories into early stage studies in patient populations, for the purposes of establishing the evidence base that will enable new therapies and diagnostics to progress to clinical practice and patient benefit at pace [22]. One condition of NIHR BRC funding is that the BRCs must have robust strategic plans in place for PPI. The NIHR University College London Hospitals Biomedical Research Centre, a partnership between UCLH and University College London, is one of the largest BRCs. The UCLH/UCL partnership has a portfolio of over 1,000 clinical research studies, opening 300 new studies every year and over 600 Principal Investigators.

Overseeing the UCLH BRC's strategy for PPI in research is a dedicated PPI team, in place to raise awareness of, and provide expertise and support for PPI in research. This article reports on one of the UCLH BRC's major PPI initiatives - an extensive programme of training workshops in PPI for researchers. The programme of workshops is just one component of a continually evolving strategy for PPI at the

For peer review only

BRC. It helps to illustrate some of the ways in which a large and complex research partnership can look to learn and innovate its research strategy.

METHODS

Training needs analysis

To inform the UCLH BRC's programme of PPI training, consultation was carried out with researchers and with UCLH patients.

1. **Needs analysis of researchers**: approximately 100 health researchers from UCL and UCLH were surveyed to ascertain educations needs and preferences. Forty-eight per cent of respondents had not previously involved patients or the public in their research although 73% reported that they intended to do so. Respondents were asked to select their training preferences from a list of topics. Over 50% of respondents selected the topics of 'How to fill in the PPI section of a funding application', 'Taster/introduction to PPI', Practical guide to planning PPI' and 'Effective partnership working with charities' as their highest preferences. Less popular topics were 'Communicating biomedical research', 'Facilitation skills', 'Chairing meetings' and 'Setting up a patient advisory group.'

2. **Consulting with patients***:* the BRC has multiple patient panels and a network of patients who work with researchers. A facilitated discussion workshop with 12 people who had previously been actively involved in working with researchers in research design was carried out. People who could not attend were asked to complete a survey on what skills they felt researchers needed training in to carry out PPI. These exercises highlighted 2 main issues for patients. First, was a sense that researchers commonly needed support in improving how they communicate with patients, for instance with more attention spent on enquiring and listening. The second was a view that researchers would benefit from greater understanding of the value of involving patients, particularly how patients could add value throughout a research project, providing practical help with the successful delivery of studies.

Patient and Public Involvement

Training was developed and carried out in partnership with patients.
Patients, who had experience of working with researchers as a part of PPI, worked with the trainers to identify and design the kind of training researchers would benefit from. This work informed the subject and format of the training workshops. This input also informed the design of the surveys of workshop attendees, enabling us to focus on the issues and skills that patients had identified as a priority. A good example is researchers' communication skills, which patients had prioritised.
Workshops were delivered with a patient and a researcher and these co-facilitators continually fed back so that workshop design could be developed and improved.

The training workshops

Drawing on the learning from the training needs analysis and discussions with patients, a programme of training workshops was established by the UCLH BRC team working closely with two very experienced, nationally-recognised trainers with specific expertise and experience in PPI in research. The programme was designed to be appealing to biomedical researchers by focusing on practical tools for PPI that

staff could deploy in their own research. The programme was designed to be flexible and iterative, the trainers working with BRC staff and patients to continually adapt the content and format of the workshops to accommodate the needs of researchers that were identified during training.

The workshop topics were 'Introduction to PPI', 'How to fill in the PPI section of a grant/REC form', 'Accessing and sustaining patients and the public', Facilitating a group discussion with patients', 'PPI Masterclass', 'PPI in early stage clinical laboratory research' and 'Effective partnership working in PPI'.

Workshops were advertised widely via the UCLH BRC website, and the organizational communication channels of UCLH, UCL, and UCL partner organisations, as well as via the BRC's social media channels. Workshops were not targeted at specific staff groups or levels of seniority. For the first 3 years of the programme, each workshop was delivered by the same 2 people – both of whom were experienced trainers. One of them is also a patient advocate. They worked closely with a researcher and a patient who helped deliver each workshop and provide additional facilitation. In the final year of the programme (2018) reported in this article, the number of workshops was rationalized to 3 topics and delivered by one member of staff from the BRC PPI team. This was because a member of staff with training experience had been appointed and this enabled a greater number of workshops to be delivered over the academic year. The workshops typically had a half day duration of between 3 and 4 hours. They were carried out at multiple different sites across UCL, UCLH and partner sites at Great Ormond Street, Moorfields Eye Hospital, UCL Partners, and Queen Mary University London. No charge was made for attendance but a small non-attendance penalty fee was introduced after year 1 of the programme to discourage non-attendance of workshops that were often significantly over-subscribed.

Workshop attendees

A total of 72 workshops were carried out over 5 years, 2014-2018. The workshops attracted 721 attendees from a variety of different professional groups and with a wide range of experience in biomedical research.

From 2018, attendees were awarded a UCL career point for every half day workshop attended.

Evaluation of the workshops

Evaluation was embedded into the design of the programme so the UCLH BRC could assess whether the workshops enabled attendees to translate their reflections and learning into research practice. Specifically,

1. Did training build up confidence and knowledge and enable researchers to carry out PPI they could not have done before?

2. What kind of PPI did they carry out and what effect did it have on research?

On arrival at the workshop, each participant was handed a survey to complete before the workshop (Survey 1) and a sealed envelope containing a survey to complete after the workshop (Survey 2) before exiting the room. An average response rate of 98% was achieved for surveys 1 and 2. Six months after the workshops, a 3rd survey (Survey 3) was sent to all participants using an online tool and a response

For peer review only

rate of 34% was achieved. Full results from Survey 3 for the 2017/18 attendees were not available for inclusion in this analysis.

Results

Workshop attendees

The workshops were attended by staff with a wide range of research roles. Of 721 attendees on the programme, data on the job roles is available on 649 (90%) staff. Thirty-one per cent of the attendees were medical consultants or scientists and 16% were research administrators and statisticians. Nearly half of the respondents listed their staff group as Other, which included a wide range of roles such as dieticians, dentists, public health specialists, physiotherapists, clinical service managers, and psychologists.

Satisfaction levels and immediate impact of the workshops on researchers

Over 95% of participants each year rated workshops very good or excellent. Workshop attendees reported marked increases in the level of awareness of the resources available to help them with PPI after attending the training. Just 17% felt they knew about the resources prior to training, rising to 80% following training. Marked increases in levels of self-reported understanding of PPI were also reported after attending training rising from 27% to 86% after training (Figure 1).

Researchers' confidence and capabilities to do PPI

Both the first and second surveys asked about attitudes, understanding and competencies in PPI, to see whether training brought about any changes. Researchers reported increased confidence and capabilities in several areas of PPI following training (figure 2). Marked increases were found in self-reported levels of confidence to do PPI, run effective meetings and to involve patients and the public in steering groups.

Impacts of the training 6 months later

The response rate for the 6 months survey was only 34% (2014-17 182/540). However, the attendees who did respond provided useful insights into the longer-term impact of the training.

Six months after their workshop 65% of the responding attendees reported that they had carried out PPI. The main areas researchers reported that patients had helped with their research in were: prioritizing research topics (45%); designing a study protocol (43%); and writing patient information materials and consent forms (36%) (figure 3).

These findings were to some extent borne out in researchers' responses to questions about the main areas where the PPI had influenced their research. Over 30% had re-written patient information materials and 36% had changed their study design. However, only 10% said their departmental/unit had made changes to research strategic direction and priorities (figure 4).

Approximately two-thirds of respondents at 6 months reported that they anticipated that PPI would ensure more meaningful outputs from their research. Nearly half predicted their research would have greater credibility with funders and stakeholders (figure 5).

For peer review only

Confidence to carry out PPI, was higher 6 months after training, rising from 58% just after training to 67% 6 months later.

Discussion

In summary, we have demonstrated that a large NIHR Biomedical Research Centre can deliver an extensive training programme in PPI in research that caters for a wide variety of biomedical research professionals at a range of levels of seniority. This included research administrators who often get overlooked but play a vital role in research funding bids and set up of studies, as well as research delivery. The preparatory scoping activities we carried out, which involved researchers and patients, helped tailor the training. Moreover, the approach of iterating workshop content enabled us to respond to the needs of researchers, ensuring workshops were relevant to the research community. The programme strategically focused on practical skills for PPI to enable researchers to build their confidence in doing PPI whilst progressively acquiring the skills to put PPI into practice in their own research, from priority setting to co-delivery of research. This focus on enabling and encouraging researchers to carry out PPI is a different emphasis to studies that recommend work that places conceptual work including power as central [23,24,25]. An important baseline finding from our work was that, prior to the training, only 17% of attendees felt they were aware of the resources available to them to support PPI in research. This finding, coupled with the fact that the training was generally well received by attendees, leads us to conclude that the workshops appeared to work well for people who had awareness that they required more skills to do PPI. Similar findings have been found in other studies of PPI in research training [18]. We are less able to draw conclusions about the benefits of the programme for people who have higher level of awareness, experience and knowledge of available resources to do PPI. Although we did introduce an experience-level distinction in the workshops in 2015 by pitching some at 'beginners' and some at 'intermediate' level, this innovation was dropped in 2018 because we found attendees tended to ignore the distinction and attendees at all workshops were of mixed experience. Further thought will be needed about how a training programme such as this can continue to evolve, building in more content and experiential learning for research staff who have greater experience of doing PPI. PPI is inherently relational. As such, the best way to learn is to 'learn by doing' and to put into practice the practical skills acquired through the type of training that the UCLH BRC has implemented. Despite, the limited response rate, the results of survey 3 would suggest first-hand experience of carrying out PPI after training helps to further increase researchers' confidence to carry out PPI. For the UCLH BRC this is a journey. We plan to continually evolve our training, and involve our researchers and patients in the programme, re-engaging researchers for their own continued learning and to enable them to share their learning with other researchers. It will also become appropriate to review the purpose of training in PPI and consider whether it is primarily to encourage and enable researchers inexperienced in PPI to involve patients and the public and discover the value of involvement, or whether there is also a need to support the more PPI-experienced researchers.

The high levels of self-reported improvements in understanding, knowledge and skills to do PPI are suggestive also of a strong benefit of the training. It is certainly true that evaluation responses focusing on levels of confidence and awareness to perform certain PPI activities recorded in the immediate aftermath of training are likely to be high as training content will still have been at the forefront of the attendee's minds at that juncture. Caution is needed not to over-interpret the findings from the 6-month survey given the low response rate and the fact that those who did respond at the 6-month stage were likely to have had more positive experience of

For peer review only

doing, or planning for, PPI in their research in the 6 months following training. Nevertheless, there are interesting signals from the data that suggest increases in confidence and knowledge in PPI were carried forward into individuals' research activities. There were also signals that carrying out PPI after training was likely to build confidence and self-reported skills. The high level of consistency in the feedback from attendees in Survey 2 (immediate) and Survey 3 (6 months) across the 5 years of the programme adds further reassurance that the positive impacts of the training that we have observed are real.

Notwithstanding the limitations of the low response rate to the 6-month survey, the findings do suggest that, following attendance at the workshops, researchers doing PPI were more likely to pursue activities at the earlier stages of the research process, notably involving patients in prioritising research topics and designing studies. The lower levels of PPI at the stage of reporting and dissemination of results may be a reflection that taking PPI into practice takes time and researchers may prefer to take forward an incremental approach to doing PPI on their new studies. This would benefit from further exploration to fully understand whether the apparent front-loading of PPI activity in the research process is real and to identify any training needs to support more PPI across the research lifecycle. The work also suggests several other areas that require further investigation, in particular the differences in impact of different kinds of involvement, and the extent to which experiential learning – learning by actually practising PPI – is key to developing PPIE in biomedical research.

One significant drawback with our evaluation is that it is based on self-report by the researchers who attended the workshops. We have not yet sought to acquire the experiential feedback of the patients who have been involved in the research activity being carried out by researchers who attended the workshops. To fully understand the impact of the training for patients it will clearly be important to incorporate patients into evaluation given the relational underpinnings of good PPI [20,21]. It will also be important to build in considerations of the quality of PPI carried out, reflecting on the use of appropriate methods for PPI [26], and on adherence to emerging standards [2,20,21]

As more funding organisations demand PPI as part of the application process, the type of training that has been developed at the UCLH BRC will be very important. During the Covid-19 pandemic, the large-scale adoption of online tools has demonstrated the effectiveness of these media for meetings and training. Further development of the programme with online training options will provide the opportunity to involve more patients and incorporate other interactive approaches, such as quizzes, in the learning process. Attention also needs to be given to overcoming the problem of self-selection in PPI, and ensuring that there is an inclusive approach to involvement in research characterised by PPI being representative of population diversity [27].

Central to the NIHR's standards for PPI is the need for researchers and research organisations to embed PPI into the culture of the organisation [2]. A training programme alone will not achieve that, particularly given the organisational complexity of major NHS-University partnerships that have Biomedical Research Centres and the consequent power dynamics that prevail within these institutions [12,28]. At the UCLH BRC, the training programme sits within a wider context in which many other PPI and engagement activities are resourced and pursued. The UCLH BRC has a dedicated, experienced and accessible team of staff who coordinate our activities in PPI and engagement and provide support and mentorship

For peer review only

for researchers. Each of the UCLH BRC's 11 scientific themes pursue theme-specific PPI activities [29]. We host a large annual Research Open Day in University College Hospital at which up to 50 research groups have displays to showcase their research for hundreds of patients and visitors to the hospital, encourage engagement with research and identify new opportunities for public involvement. We fund 50 laboratory placements with UCL biomedical scientists every year for school pupils from disadvantaged backgrounds [30]. We also work with researchers to develop innovative ways to communicate research with language and formats that are accessible. This package of broader support and resources helps build researchers' confidence and skill levels to do PPI alongside the BRC's structured training programme in PPI.

The on-going challenge is to weave all of these complementary initiatives together to drive positive change, and high-quality PPI, in a large community of biomedical researchers. The extent to which these activities actually change researchers' practice is not easy to measure, especially in a large complex biomedical research partnership. The hope is that, within a broad and varied approach to PPI, such as that at the UCLH BRC, researchers will find things to inspire them to continue to explore good ways to involve patients in their research.

Key recommendations

- Training of researchers should be considered a fundamental part of developing the involvement of the public in research and built into long-term strategic planning and investment.
- Training should be tailored to give researchers practical skills, building up their confidence, practical knowledge and the capacity to experience firsthand the value of PPI to research. This pragmatic approach can lay the foundations for the future by equipping a generation of researchers to involve patients and the public in research.
- Further evaluation is recommended to understand how beneficial to research PPI training is, or indeed whether patients perceive any impact of PPI on research.

Contributorship statement

Rosamund M Yu (corresponding and lead author and guarantor) lead contribution: established and managed the training programme, designed the evaluation of the programme and carried out the analysis of findings, and wrote the first and part of the final draft of the article.

Simon Denegri equal contribution: leader of the work area and advised on production of the training, and its analysis, and edited and steered the article from draft to final stage.

Bec Hanley equal contribution: one of the trainers commissioned, with another trainer, to design and carry out the training, including its evaluation, and edited and steered the article from draft to final stage.

Jaber Ahmed equal contribution: carried out analysis of statistical findings.

Nicholas J McNally equal contribution: led and oversaw the Biomedical Research Centre team carrying out the training programme and managed its final outcome, and steered and edited the article from draft to final stage. Wrote the final draft of the manuscript.

For peer review only

Acknoweldgement
Special thanks to patient advisors Libby Cooper, Brenden Conroy, Philip Creasy, Anthony Locke.

Competing interests

All authors have completed the Unified Competing Interest form and, with 2 exceptions, declare: no support from any organisation for the submitted work; no financial relationships with any organisations that might have an interest in the submitted work in the previous three years], no other relationships or activities that could appear to have influenced the submitted work]. One author discloses that they were a paid employee of the institution running the training discussed at the time this work was undertaken. Another author discloses that they received a fee to deliver the training discussed in the article.

Ethical approval and study sponsors

This work is an evaluation of a training programme and is not research. As such, Research Ethics Committee approval was not sought for the evaluation.

Funding

National Institute for Health Research Biomedical Research Centre at University College London Hospitals NHS Foundation Trust
Health Education North Central and East London
Wellcome grant number 204841/Z/16/Z.

Data Sharing Statement

Data is made up of answers to surveys by researchers attending training. Attendees are not identifiable. Data is not in a repository and can be requested from Rosamund.yu@ucl.ac.uk

Figure captions

Figure 1 Knowledge and understanding of PPI
Figure 2 PPI skills
Figure 3 Kinds of PPI carried out
Figure 4 Impact of PPI on research
Figure 5 Anticipated future impact of PPI on research

References

1.NIHR. Going the extra mile: improving the nation’s health and wellbeing through public involvement in research. Eastleigh: NIHR; 2015.

2. NIHR. UK standards for public involvement. NIHR; 2019

3. Domecq JP, Prutsky G, Elraiyah T, Wang Z, Nabhan M, Shippee N, et al. Patient engagement in research: a systematic review. BMC Health Serv Res. 2014;14:89.

4. Brett J, Staniszewska S, Mockford C, Herron-Marx S, Hughes J, Tysall C, et al. Mapping the impact of patient and public involvement on health and social care research: a systematic review. Health Expect. 2014;17(5):637–50.

For peer review only

5. Staley K. Exploring impact: public involvement in NHS, public health and social care research. INVOLVE. Eastleigh: National Institute of Health Research, Research NIoH; 2009.

6. Brett J, Staniszewska S, Mockford C, Herron-Marx S, Hughes J, Tysall C, et al. A systematic review of the impact of patient and public involvement on service users, researchers and communities. Patient. 2014;7(4):387–95.

7. Patterson S, Trite J, Weaver T. Activity and views of service users involved in mental health research: UK survey. Br J Psychiatry. 2014;205(1):68–75.

8. Green G, Johns T. Exploring the relationship (and Power Dynamic) between researchers and public partners working together in applied health research teams. Front Sociol. 2019;4(20)..

9. O'Shea A, Boaz AL, Chambers M. A hierarchy of power: The place of patient and public involvement in healthcare service development. Front Sociol. 2019;4:38.

10. Boaz A, Biri D, McKevitt C. Rethinking the relationship between science and society: has there been a shift in attitudes to patient and public involvement and public engagement in science in the United Kingdom? Health Expect. 2016;19(3):592–601.

11. Romsland GI, Milosavljevic KL, Andreassen TA. Facilitating non-tokenistic user involvement in research. Res Involv Engagem. 2019;5(1):18.

12. Evans J, Papoulias C. Between funder requirements and 'jobbing scientists': the evolution of patient and public involvement in a mental health biomedical research centre - a qualitative study. Research Involvement and Engagement. 2020;6-12

13. Boylan A-M, Locock L, Staniszewska S. "About sixty per cent I want to do it": Health researchers' attitudes to, and experiences of, patient and public involvement (PPI) – A qualitative study.

14. INVOLVE survey of priorities for public involvement across the NIHR September 2011

15. Concordat for Engaging the Public with Research

16. Thompson J, Barber R, Ward PR, Boote JD, Cooper CL, Armitage CJ, et al. Health researchers' attitudes towards public involvement in health research. Health Expect 12(2):209-20 (2009).

17. McPin Foundation – Kirsten Morgan. Talking Point Papers 2: Patient and Public Involvement in Research- why not? 2016 https://mcpin.org/wp-content/uploads/talking-point-paper-2.pdf

18. McKenzie, A., Alpers, K., Heyworth, J. *et al.* Consumer and community involvement in health and medical research: evaluation by online survey of Australian training workshops for researchers. *Res Involv Engagem* 2016 **2,** 16

19. Dudley, L., Gamble, C., Allam, A. *et al.* A little more conversation please? Qualitative study of researchers' and patients' interview accounts of training for

For peer review only

patient and public involvement in clinical trials. *Trials* **16,** 190 (2015) doi:10.1186/s13063-015-0667-4

20. Staniszewska S, Brett J, Simera I, Seers K, Mockford C, Goodlad S, Altman DG, Moher D, Barber R, Denegri S, Entwistle A. GRIPP2 reporting checklists: tools to improve reporting of patient and public involvement in research. BMJ. 2017 Aug 2;358.

21. Brett J, Staniszewska S, Simera I, Seers K, Mockford C, Goodlad S, Altman D, Moher D, Barber R, Denegri S, Entwistle AR. Reaching consensus on reporting patient and public involvement (PPI) in research: methods and lessons learned from the development of reporting guidelines. BMJ open. 2017 Oct 1;7(10):e016948.

22. NIHR. Experimental medicine. https://www.nihr.ac.uk/explore-nihr/support/experimental-medicine.htm

23. Martin GP. 'Ordinary people only': knowledge, representativeness, and the publics of public participation in healthcare. Sociology of health & illness. 2008 Jan;30(1):35-54.

24. Beresford P. User involvement in research: exploring the challenges. NT Res. 2003;8(1):36–46.

25. Beresford P. Public participation in health and social care: Exploring the co-production of knowledge. Front Sociol. 2019;3:41.

26. Doria N, Condran B, Boulos L, Maillet DG, Dowling L, Levy A. Sharpening the focus: differentiating between focus groups for patient engagement vs. qualitative research. Research Involvement and Engagement. 2018 Dec;4(1):1-8.

27. Boote J, Telford R, Cooper C. Consumer involvement in health research: a review and research agenda. Health Policy 61:213-36 (2002).

28. Petit-Zeman S. What do researchers really think about PPI? 2016. NIHR Oxford Biomedical Research Centre (2016) https://oxfordbrc.nihr.ac.uk/blog/what-do-researchers-really-think-about-ppi/

29. Crutch S, Herron D, Pickett J, Rosser S, Rossor M. Inspired by chance: valuing patients' informal contributions to research. BMJ. 2020 Dec 14;371.

30. In2Science. In2Science UK Impact Report 2020. https://in2scienceuk.org/wp-content/uploads/2020/11/In2scienceUK-Impact-Report-2020-digital.pdf

For peer review only

Figure 1

Knowledge and understanding

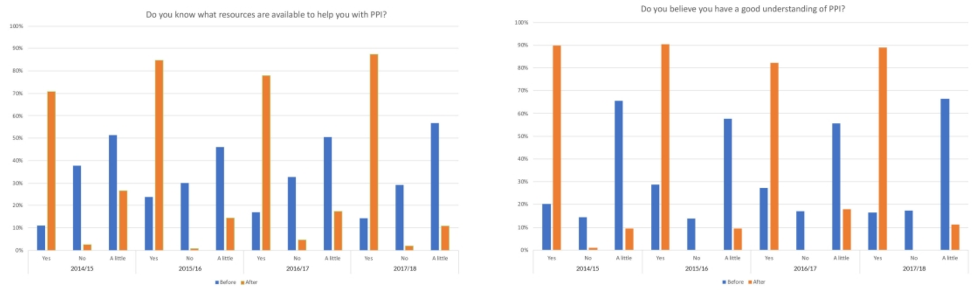

Figure 1: understanding and knowledge

296x142mm (300 x 300 DPI)

Figure 2

PPI skills - what researchers feel able to do before and after training (surveys 1, 2 and 3)

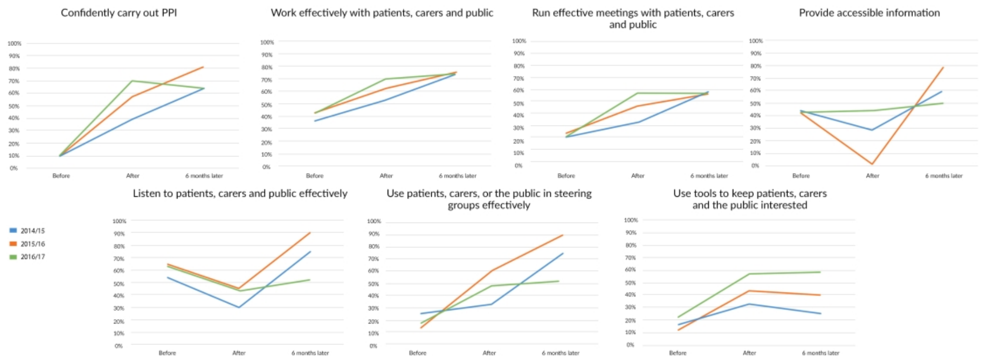

Figure 2: PPI skills - what researchers feel able to do before and after training (surveys 1,2 and3)

297x147mm (300 x 300 DPI)

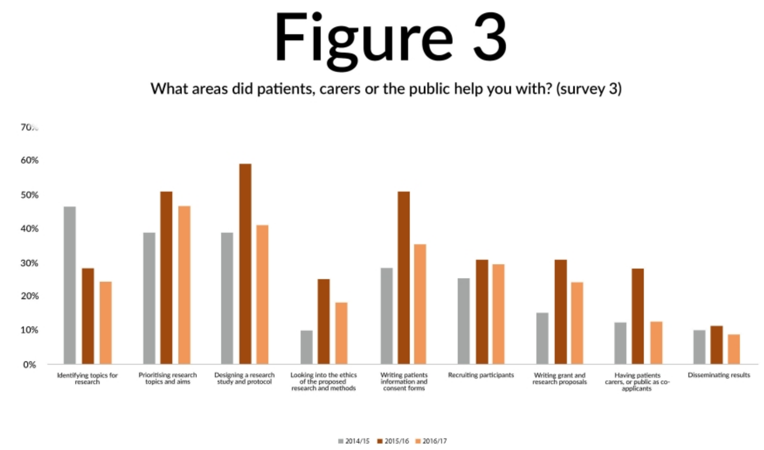

Figure 3: What areas did patients, carers or the public help you with (survey 3)

297x147mm (300 x 300 DPI)

Figure 4

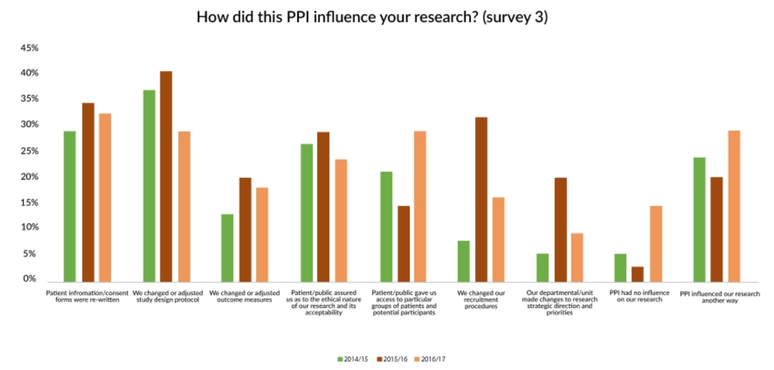

Figure 4: How did this PPI influence your research (survey3)

297x147mm (300 x 300 DPI)

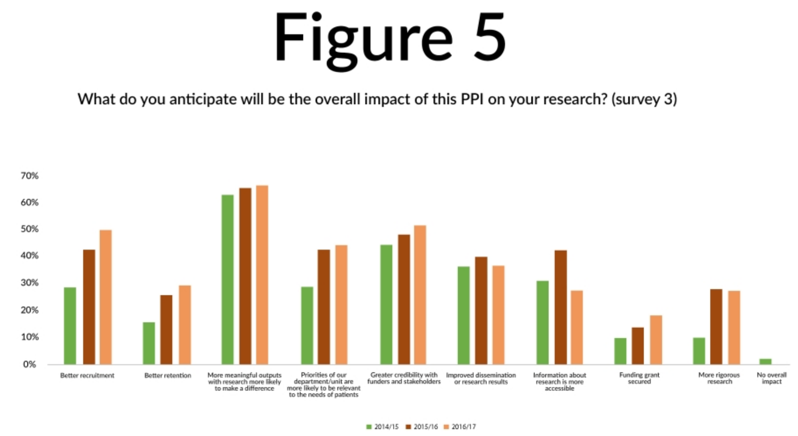

Figure 5: What do you anticipate will be the overall impact of this PPI on your research? (survey 3)

297x147mm (300 x 300 DPI)

BMJ Open

Evaluation of a patient and public involvement training programme for researchers at a large biomedical research centre in the United Kingdom.

Journal:	*BMJ Open*
Manuscript ID	bmjopen-2020-047995.R2
Article Type:	Original research
Date Submitted by the Author:	01-Jun-2021
Complete List of Authors:	Yu, Rosamund; University College London Hospitals NHS Foundation Trust, NIHR biomedical research centre at UCLH<br>Hanley , Bec ; Medical Research Council Clinical Trials Unit at University College London<br>Denegri, Simon; Academy of Medical Sciences<br>Ahmed, Jaber; University College London Hospitals NHS Foundation Trust<br>McNally, Nicholas; University College London
**Primary Subject Heading**:	Research methods
Secondary Subject Heading:	Patient-centred medicine, Medical education and training
Keywords:	EDUCATION & TRAINING (see Medical Education & Training), Clinical governance < HEALTH SERVICES ADMINISTRATION & MANAGEMENT, Organisational development < HEALTH SERVICES ADMINISTRATION & MANAGEMENT, MEDICAL EDUCATION & TRAINING, STATISTICS & RESEARCH METHODS

SCHOLARONE™
Manuscripts

I, the Submitting Author has the right to grant and does grant on behalf of all authors of the Work (as defined in the below author licence), an exclusive licence and/or a non-exclusive licence for contributions from authors who are: i) UK Crown employees; ii) where BMJ has agreed a CC-BY licence shall apply, and/or iii) in accordance with the terms applicable for US Federal Government officers or employees acting as part of their official duties; on a worldwide, perpetual, irrevocable, royalty-free basis to BMJ Publishing Group Ltd ("BMJ") its licensees and where the relevant Journal is co-owned by BMJ to the co-owners of the Journal, to publish the Work in this journal and any other BMJ products and to exploit all rights, as set out in our licence.

The Submitting Author accepts and understands that any supply made under these terms is made by BMJ to the Submitting Author unless you are acting as an employee on behalf of your employer or a postgraduate student of an affiliated institution which is paying any applicable article publishing charge ("APC") for Open Access articles. Where the Submitting Author wishes to make the Work available on an Open Access basis (and intends to pay the relevant APC), the terms of reuse of such Open Access shall be governed by a Creative Commons licence – details of these licences and which Creative Commons licence will apply to this Work are set out in our licence referred to above.

Other than as permitted in any relevant BMJ Author's Self Archiving Policies, I confirm this Work has not been accepted for publication elsewhere, is not being considered for publication elsewhere and does not duplicate material already published. I confirm all authors consent to publication of this Work and authorise the granting of this licence.

For peer review only

Title: Evaluation of a patient and public involvement training programme for researchers at a large biomedical research centre in the United Kingdom.

Authors

Rosamund M Yu (corresponding and lead author and guarantor)

Rosamund.yu@ucl.ac.uk
Head of PPI and Research Communications, National Institue for Health Research Biomedical Research Centre at University College London Hospitals NHS Foundation Trust
1st Floor, Suite A, Maple House, 149 Tottenham Court Road, London W1T 7DN
Tel: 07962943966
Fax: 020 7380 9937

Bec Hanley
Freelance adviser on patient involvement
National Institute for Health Research Biomedical Research Centre at University College London Hospitals NHS Foudnation Trust
1st Floor, Suite A, Maple House, 149 Tottenham Court Road, London W1T 7DN, UK

Simon Denegri
Executive Director, Academy of Medical Sciences
41 Portland Place
London W1B 1QH, UK

Jaber Ahmed
Digital Communications Officer National Institue for Health Research Biomedical Research Centre at University College London Hospitals NHS Foudnation Trust
1st Floor, Suite A, Maple House, 149 Tottenham Court Road, London W1T 7DN
Contribution: Author carried out analysis of statistical findings.

Nicholas J McNally
Managing Director, Research UCLH/UCL
National Institute for Health Research Biomedical Research Centre at University College London Hospitals NHS Foudnation Trust
1st Floor, Suite A, Maple House, 149 Tottenham Court Road, London W1T 7DN, UK

Key words

Patient Involvement

Research personnel
Biomedical research
Continuing medical education

Word count – (excluding title page, references, figures tables, contributorship, competing interest and data statements): 3903

For peer review only

Abstract

Objectives: To design, deliver and evaluate a programme of training workshops for biomedical researchers aimed at building confidence and skills in actively involving patients and the public (PPI) in research.

Design: A bespoke programme of training workshops in patient and public involvement aimed at researchers.

Setting: A large National Institute for Health Research Biomedical Research Centre in London and several partner organisations.

Participants: 721 scientists, clinicians and research managers attending dedicated training in patient and public involvement at a major London NHS-university partnership.

Interventions: A programme of 72 training workshops, designed to build practical skills and confidence for researchers working with patients and the public in research, was delivered at a major research-active NHS:university partnership. An iterative approach was taken to the programme, with the content of the workshops continually reviewed and refreshed to respond to the needs of researchers. Surveys before, immediately following and 6 months after training investigated the impact on researchers' confidence and skills in PPI work, and the kind of PPI they subsequently carried out.

Results: Training brought about immediate marked increases in researchers' self-reported confidence to carry out PPI activities within their research, and in their knowledge of good practice. The evaluation indicates that workshop attendees were more likely to involve patients in their research following training. Researchers tended to involve patients and the public in a range of areas, including input to study design and patient information, in particular.

Conclusions: When positioned within a broader organisational strategy for PPI in research, such training has an important role to play in progressing PPI in a major research partnership. Training appeared to provide the confidence needed to carry out PPI which enabled further development of confidence and skills. Involving researchers who have been trained in the on-going development of the training programme and bringing in patients to the on-going training programme are key next steps.

Strengths and Limitations

- This study evaluates probably one of the largest PPI in biomedical research training programmes in the UK, carried out across a major research partnership.
- The precise content of the training workshops was iterative and evolved throughout the programme in response to feedback from participants, making it highly tailored to researchers' needs
- The evaluation was carefully designed to monitor the impact of PPI training on researchers and their work. However, It has not sought the perspectives of patients participating in projects being carried out by the researchers.
- Response rates to the surveys carried out immediately before and after training were high (average 98%). However, response rates to the survey carried out at 6 months post-training were low (34%). Respondents were self-selecting and their responses were dependent on researchers' self-evaluation and recall.

For peer review only

INTRODUCTION

In the UK, patient and public involvement (PPI) in biomedical research has been encouraged and promoted over the last 15 years. Evidence of PPI has become a condition of many research funders, notably the National Institute for Health Research (NIHR), which has helped to set expectations for PPI [1]. The NIHR's standards for public involvement are designed to improve quality and consistency of PPI in health research, emphasizing the importance of inclusive opportunities, working together, support and learning, communications, impact and governance, as values-based areas for reflection and learning for researchers and research organisations [2].

There are two dominant narratives underpinning the importance of patient and public involvement in biomedical research. First is the notion that PPI improves quality, relevance and acceptability of research [3,4,5]. The second is that PPI enables citizens to exercise their rights. This can mean that they feel empowered and valued and that they can build their skills and/or knowledge. [6,7]. The power dynamic that exists between healthcare professionals, researchers and patients can be deep rooted [8,9,10]. The primacy that is given to clinical or scientific knowledge over the experiential knowledge that patients bring to the research process has been shown to render much involvement practice as tokenistic [11]. There are considerable challenges of establishing meaningful PPI in hierarchical, scientific research organisational settings [12].

Researchers' experiences and attitudes towards PPI are undoubtedly key to embedding involvement within the wider research culture of a research organisation [13]. Dedicated training in PPI has long been identified as a need [14] and an important mechanism for developing researchers' skills, experiences and attitudes to PPI [15,16,17,18,19]. Researchers are also being challenged to document in a structured way how they involve patients throughout the research process to ensure PPI practice is based on the best evidence [20,21].

In England, the National Institute for Health Research Biomedical Research Centres (BRCs) are partnerships between NHS Trusts and universities. The BRCs are funded by the NIHR to drive experimental medicine research, taking promising scientific concepts from laboratories into early stage studies in patient populations, for the purposes of establishing the evidence base that will enable new therapies and diagnostics to progress to clinical practice and patient benefit at pace [22]. One condition of NIHR BRC funding is that the BRCs must have robust strategic plans in place for PPI. The NIHR University College London Hospitals Biomedical Research Centre, a partnership between UCLH and University College London, is one of the largest BRCs. The UCLH/UCL partnership has a portfolio of over 1,000 clinical research studies, opening 300 new studies every year and over 600 Principal Investigators.

Overseeing the UCLH BRC's strategy for PPI in research is a dedicated PPI team, in place to raise awareness of, and provide expertise and support for PPI in research. This article reports on one of the UCLH BRC's major PPI initiatives - an extensive programme of training workshops in PPI for researchers. The programme of workshops is just one component of a continually evolving strategy for PPI at the BRC. It helps to illustrate some of the ways in which a large and complex research partnership can look to learn and innovate its research strategy. This article reports on how the UCLH BRC has, developed and deployed an extensive training programme in PPI for research staff as a major component embedding a more extensive culture of PPI in research.

For peer review only

METHODS

Training needs analysis

To inform the UCLH BRC's programme of PPI training, consultation was carried out with researchers and with UCLH patients.

1. **Needs analysis of researchers**: approximately 100 health researchers from UCL and UCLH were surveyed to ascertain educations needs and preferences. Forty-eight per cent of respondents had not previously involved patients or the public in their research although 73% reported that they intended to do so. Respondents were asked to select their training preferences from a list of topics. Over 50% of respondents selected the topics of 'How to fill in the PPI section of a funding application', 'Taster/introduction to PPI', Practical guide to planning PPI' and 'Effective partnership working with charities' as their highest preferences. Less popular topics were 'Communicating biomedical research', 'Facilitation skills', 'Chairing meetings' and 'Setting up a patient advisory group.'

2. **Consulting with patients*:*** the BRC has multiple patient panels and a network of patients who work with researchers. A facilitated discussion workshop with 12 people who had previously been actively involved in working with researchers in research design was carried out. People who could not attend were asked to complete a survey on what skills they felt researchers needed training in to carry out PPI. These exercises highlighted 2 main issues for patients. First, was a sense that researchers commonly needed support in improving how they communicate with patients, for instance with more attention spent on enquiring and listening. The second was a view that researchers would benefit from greater understanding of the value of involving patients, particularly how patients could add value throughout a research project, providing practical help with the successful delivery of studies.

3. **Patient and Public Involvement**
   Training was developed and carried out in partnership with patients. Patients, who had experience of working with researchers as a part of PPI, worked with the trainers to identify and design the kind of training researchers would benefit from. This work informed the subject and format of the training workshops. This input also informed the design of the surveys of workshop attendees, enabling us to focus on the issues and skills that patients had identified as a priority. A good example is researchers' communication skills, which patients had highlighted.
   Workshops were delivered with a patient and a researcher and these co-facilitators continually fed back so that workshop design could be developed and improved.

The training workshops

Drawing on the learning from the training needs analysis and discussions with patients, a programme of training workshops was established by the UCLH BRC team working closely with two very experienced, nationally-recognised trainers with specific expertise and experience in PPI in research. The programme was designed to be appealing to biomedical researchers by focusing on practical tools for PPI that

For peer review only - http://bmjopen.bmj.com/site/about/guidelines.xhtml

staff could deploy in their own research. The programme was designed to be flexible and iterative, the trainers working with BRC staff and patients to continually adapt the content and format of the workshops to accommodate the needs of researchers that were identified during training.

The workshop topics were ‘Introduction to PPI’, ‘How to fill in the PPI section of a grant/REC form’, ‘Accessing and sustaining patients and the public’, Facilitating a group discussion with patients’, ‘PPI Masterclass’, ‘PPI in early stage clinical laboratory research’ and ‘Effective partnership working in PPI’.

Workshops were advertised widely via the UCLH BRC website, and the organizational communication channels of UCLH, UCL, and UCL partner organisations, as well as via the BRC’s social media channels. Workshops were not targeted at specific staff groups or levels of seniority. For the first 3 years of the programme, each workshop was delivered by the same 2 people – both of whom were experienced trainers. One of them is also a patient advocate. They worked closely with a researcher and a patient who helped deliver each workshop and provide additional facilitation. In the final year of the programme (2018) reported in this article, the number of workshops was rationalized to 3 topics and delivered by one member of staff from the BRC PPI team. This was because a member of staff with training experience had been appointed and this enabled a greater number of workshops to be delivered over the academic year. The workshops typically had a half day duration of between 3 and 4 hours. They were carried out at multiple different sites across UCL, UCLH and partner sites at Great Ormond Street, Moorfields Eye Hospital, UCL Partners, and Queen Mary University London. No charge was made for attendance but a small non-attendance penalty fee was introduced after year 1 of the programme to discourage non-attendance of workshops that were often significantly over-subscribed.

Workshop attendees

A total of 72 workshops were carried out over 5 years, 2014-2018. The workshops attracted 721 attendees from a variety of different professional groups and with a wide range of experience in biomedical research.

From 2018, attendees were awarded a UCL career point for every half day workshop attended.

Evaluation of the workshops

Evaluation was embedded into the design of the programme so the UCLH BRC could assess whether the workshops enabled attendees to translate their reflections and learning into research practice. Specifically,

1. Did training build up confidence and knowledge and enable researchers to carry out PPI they could not have done before?

2. What kind of PPI did they carry out and what effect did it have on research?

On arrival at the workshop, each participant was handed a survey to complete before the workshop (Survey 1) and a sealed envelope containing a survey to complete after the workshop (Survey 2) before exiting the room. An average response rate of 98% was achieved for surveys 1 and 2. Six months after the workshops, a 3rd survey (Survey 3) was sent to all participants using an online tool and a response rate of 34% was achieved. Full results from Survey 3 for the 2017/18 attendees were

For peer review only

not available for inclusion in this analysis. The survey questions can be viewed in the Measures supplement

Data Analysis

All evaluation data was entered into Microsoft Excel where descriptive analysis was carried out.

Results

Workshop attendees

The workshops were attended by staff with a wide range of research roles. Of 721 attendees on the programme, data on the job roles is available on 649 (90%) staff. Thirty-one per cent of the attendees were medical consultants or scientists and 16% were research administrators and statisticians. Nearly half of the respondents listed their staff group as Other, which included a wide range of roles such as dieticians, dentists, public health specialists, physiotherapists, clinical service managers, and psychologists.

Satisfaction levels and immediate impact of the workshops on researchers

Over 95% of participants each year rated workshops very good or excellent. Workshop attendees reported marked increases in the level of awareness of the resources available to help them with PPI after attending the training. Just 17% felt they knew about the resources prior to training, rising to 80% following training. Marked increases in levels of self-reported understanding of PPI were also reported after attending training rising from 27% to 86% after training (Figure 1).

Researchers' confidence and capabilities to do PPI

Both the first and second surveys asked about attitudes, understanding and competencies in PPI, to see whether training brought about any changes. Researchers reported increased confidence and capabilities in several areas of PPI following training (figure 2). Marked increases were found in self-reported levels of confidence to do PPI, run effective meetings and to involve patients and the public in steering groups.

Impacts of the training 6 months later

The response rate for the 6 months survey was only 34% (2014-17 182/540). However, the attendees who did respond provided useful insights into the longer-term impact of the training.

Six months after their workshop 65% of the responding attendees reported that they had carried out PPI. The main areas researchers reported that patients had helped with their research in were: prioritizing research topics (45%); designing a study protocol (43%); and writing patient information materials and consent forms (36%) (figure 3).

These findings were to some extent borne out in researchers' responses to questions about the main areas where the PPI had influenced their research. Over 30% had re-written patient information materials and 36% had changed their study design. However, only 10% said their departmental/unit had made changes to research strategic direction and priorities (figure 4).

For peer review only

Approximately two-thirds of respondents at 6 months reported that they anticipated that PPI would ensure more meaningful outputs from their research. Nearly half predicted their research would have greater credibility with funders and stakeholders (figure 5).

Confidence to carry out PPI, was higher 6 months after training, rising from 58% just after training to 67% 6 months later.

Discussion

In summary, we have demonstrated that a large NIHR Biomedical Research Centre can deliver an extensive training programme in PPI in research that caters for a wide variety of biomedical research professionals at a range of levels of seniority. This included research administrators who often get overlooked but play a vital role in research funding bids and set up of studies, as well as research delivery. The preparatory scoping activities we carried out, which involved researchers and patients, helped tailor the training. Moreover, the approach of iterating workshop content enabled us to respond to the needs of researchers, ensuring workshops were relevant to the research community. The programme strategically focused on practical skills for PPI to enable researchers to build their confidence in doing PPI whilst progressively acquiring the skills to put PPI into practice in their own research, from priority setting to co-delivery of research. This focus on enabling and encouraging researchers to carry out PPI is a different emphasis to studies that recommend work that places conceptual work including power as central [23,24,25]. An important baseline finding from our work was that, prior to the training, only 20% of attendees felt they were aware of the resources available to them to support PPI in research. This finding, coupled with the fact that the training was generally well received by attendees, leads us to conclude that the workshops appeared to work well for people who had awareness that they required more skills to do PPI. Similar findings have been found in other studies of PPI in research training [18]. We are less able to draw conclusions about the benefits of the programme for people who have higher level of awareness, experience and knowledge of available resources to do PPI. Although we did introduce an experience-level distinction in the workshops in 2015 by pitching some at 'beginners' and some at 'intermediate' level, this innovation was dropped in 2018 because we found attendees tended to ignore the distinction and attendees at all workshops were of mixed experience. Further thought will be needed about how a training programme such as this can continue to evolve, building in more content and experiential learning for research staff who have greater experience of doing PPI. PPI is inherently relational. As such, the best way to learn is to 'learn by doing' and to put into practice the practical skills acquired through the type of training that the UCLH BRC has implemented. Despite, the limited response rate, the results of survey 3 would suggest first-hand experience of carrying out PPI after training helps to further increase researchers' confidence to carry out PPI. For the UCLH BRC this is a journey. We plan to continually evolve our training, and involve our researchers and patients in the programme, re-engaging researchers for their own continued learning and to enable them to share their learning with other researchers. It will also become appropriate to review the purpose of training in PPI and consider whether it is primarily to encourage and enable researchers inexperienced in PPI to involve patients and the public and discover the value of involvement, or whether there is also a need to support the more PPI-experienced researchers.

The high levels of self-reported improvements in understanding, knowledge and skills to do PPI are suggestive also of a strong benefit of the training. It is certainly true that evaluation responses focusing on levels of confidence and awareness to

For peer review only - http://bmjopen.bmj.com/site/about/guidelines.xhtml

perform certain PPI activities recorded in the immediate aftermath of training are likely to be high as training content will still have been at the forefront of the attendee's minds at that juncture. Caution is needed not to over-interpret the findings from the 6-month survey given the low response rate and the fact that those who did respond at the 6-month stage were likely to have had more positive experience of doing, or planning for, PPI in their research in the 6 months following training. Nevertheless, there are interesting signals from the data that suggest increases in confidence and knowledge in PPI were carried forward into individuals' research activities. There were also signals that carrying out PPI after training was likely to build confidence and self-reported skills. The high level of consistency in the feedback from attendees in Survey 2 (immediate) and Survey 3 (6 months) across the 5 years of the programme adds further reassurance that the positive impacts of the training that we have observed are real.

Notwithstanding the limitations of the low response rate to the 6-month survey, the findings do suggest that, following attendance at the workshops, researchers doing PPI were more likely to pursue activities at the earlier stages of the research process, notably involving patients in prioritising research topics and designing studies. The lower levels of PPI at the stage of reporting and dissemination of results may be a reflection that taking PPI into practice takes time and researchers may prefer to take forward an incremental approach to doing PPI on their new studies. This would benefit from further exploration to fully understand whether the apparent front-loading of PPI activity in the research process is real and to identify any training needs to support more PPI across the research lifecycle. The work also suggests several other areas that require further investigation, in particular the differences in impact of different kinds of involvement, and the extent to which experiential learning – learning by actually practising PPI – is key to developing PPIE in biomedical research.

One significant drawback with our evaluation is that it is based on self-report by the researchers who attended the workshops. We have not yet sought to acquire the experiential feedback of the patients who have been involved in the research activity being carried out by researchers who attended the workshops. To fully understand the impact of the training for patients it will clearly be important to incorporate patients into evaluation given the relational underpinnings of good PPI [20,21]. It will also be important to build in considerations of the quality of PPI carried out, reflecting on the use of appropriate methods for PPI [26], and on adherence to emerging standards [2,20,21]

As more funding organisations demand PPI as part of the application process, the type of training that has been developed at the UCLH BRC will be very important. During the Covid-19 pandemic, the large-scale adoption of online tools has demonstrated the effectiveness of these media for meetings and training. Further development of the programme with online training options will provide the opportunity to involve more patients and incorporate other interactive approaches, such as quizzes, in the learning process. Attention also needs to be given to overcoming the problem of self-selection in PPI, and ensuring that there is an inclusive approach to involvement in research characterised by PPI being representative of population diversity [27].

Central to the NIHR's standards for PPI is the need for researchers and research organisations to embed PPI into the culture of the organisation [2]. A training programme alone will not achieve that, particularly given the organisational complexity of major NHS-University partnerships that have Biomedical Research

For peer review only

Centres and the consequent power dynamics that prevail within these institutions [12,28]. At the UCLH BRC, the training programme sits within a wider context in which many other PPI and engagement activities are resourced and pursued. The UCLH BRC has a dedicated, experienced and accessible team of staff who coordinate our activities in PPI and engagement and provide support and mentorship for researchers. Each of the UCLH BRC's 11 scientific themes pursue theme-specific PPI activities [29]. We host a large annual Research Open Day in University College Hospital at which up to 50 research groups have displays to showcase their research for hundreds of patients and visitors to the hospital, encourage engagement with research and identify new opportunities for public involvement. We fund 50 laboratory placements with UCL biomedical scientists every year for school pupils from disadvantaged backgrounds [30]. We also work with researchers to develop innovative ways to communicate research with language and formats that are accessible. This package of broader support and resources helps build researchers' confidence and skill levels to do PPI alongside the BRC's structured training programme in PPI.

The on-going challenge is to weave all of these complementary initiatives together to drive positive change, and high-quality PPI, in a large community of biomedical researchers. The extent to which these activities actually change researchers' practice is not easy to measure, especially in a large complex biomedical research partnership. The hope is that, within a broad and varied approach to PPI, such as that at the UCLH BRC, researchers will find things to inspire them to continue to explore good ways to involve patients in their research.

Key recommendations

- Training of researchers should be considered a fundamental part of developing the involvement of the public in research and built into long-term strategic planning and investment.
- Training should be tailored to give researchers practical skills, building up their confidence, practical knowledge and the capacity to experience firsthand the value of PPI to research. This pragmatic approach can lay the foundations for the future by equipping a generation of researchers to involve patients and the public in research.
- Further evaluation is recommended to understand how beneficial to research PPI training is, or indeed whether patients perceive any impact of PPI on research.

Contributorship statement

Rosamund M Yu (corresponding and lead author and guarantor) lead contribution: established and managed the training programme, designed the evaluation of the programme and carried out the analysis of findings, and wrote the first and part of the final draft of the article.

Nicholas J McNally equal contribution: led and oversaw the Biomedical Research Centre team carrying out the training programme and managed its final outcome, and steered and edited the article from draft to final stage. Wrote the final draft of the manuscript.

Simon Denegri equal contribution: leader of the work area and advised on production of the training, and its analysis, and edited and steered the article from draft to final stage.

For peer review only

Bec Hanley equal contribution: one of the trainers commissioned, with another trainer, to design and carry out the training, including its evaluation, and edited and steered the article from draft to final stage.

Jaber Ahmed equal contribution: carried out analysis of statistical findings.

Special thanks to patient advisors Libby Cooper, Brenden Conroy, Philip Creasy, Anthony Locke.

Competing interests

All authors have completed the Unified Competing Interest form and, with 2 exceptions, declare: no support from any organisation for the submitted work; no financial relationships with any organisations that might have an interest in the submitted work in the previous three years], no other relationships or activities that could appear to have influenced the submitted work]. One author discloses that they were a paid employee of the institution running the training discussed at the time this work was undertaken. Another author discloses that they received a fee to deliver the training discussed in the article.

Ethical approval and study sponsors

This work is an evaluation of a training programme and is not research. As such, Research Ethics Committee approval was not sought for the evaluation.

Funding

National Institue for Health Research Biomedical Research Centre at University College London Hospitals NHS Foundation Trust - Award/Grant number is not applicable
Health Education North Central and East London – grant WFDevProjects062
Wellcome - grant number 204841/Z/16/Z.

Data Sharing Statement

Data is made up of answers to surveys by researchers attending training. Attendees are not identifiable. Data is not in a repository and can be requested from Rosamund.yu@ucl.ac.uk

Figure captions

Figure 1 Knowledge and understanding of PPI
Figure 2 PPI skills
Figure 3 Kinds of PPI carried out
Figure 4 Impact of PPI on research
Figure 5 Anticipated future impact of PPI on research

References

1.NIHR. Going the extra mile: improving the nation's health and wellbeing through public involvement in research. Eastleigh: NIHR; 2015.

2. NIHR. UK standards for public involvement. NIHR; 2019

3. Domecq JP, Prutsky G, Elraiyah T, Wang Z, Nabhan M, Shippee N, et al. Patient engagement in research: a systematic review. BMC Health Serv Res. 2014;14:89.

For peer review only

4. Brett J, Staniszewska S, Mockford C, Herron-Marx S, Hughes J, Tysall C, et al. Mapping the impact of patient and public involvement on health and social care research: a systematic review. Health Expect. 2014;17(5):637–50.

5. Staley K. Exploring impact: public involvement in NHS, public health and social care research. INVOLVE. Eastleigh: National Institute of Health Research, Research NIoH; 2009.

6. Brett J, Staniszewska S, Mockford C, Herron-Marx S, Hughes J, Tysall C, et al. A systematic review of the impact of patient and public involvement on service users, researchers and communities. Patient. 2014;7(4):387–95.

7. Patterson S, Trite J, Weaver T. Activity and views of service users involved in mental health research: UK survey. Br J Psychiatry. 2014;205(1):68–75.

8. Green G, Johns T. Exploring the relationship (and Power Dynamic) between researchers and public partners working together in applied health research teams. Front Sociol. 2019;4(20)..

9. O'Shea A, Boaz AL, Chambers M. A hierarchy of power: The place of patient and public involvement in healthcare service development. Front Sociol. 2019;4:38.

10. Boaz A, Biri D, McKevitt C. Rethinking the relationship between science and society: has there been a shift in attitudes to patient and public involvement and public engagement in science in the United Kingdom? Health Expect. 2016;19(3):592–601.

11. Romsland GI, Milosavljevic KL, Andreassen TA. Facilitating non-tokenistic user involvement in research. Res Involv Engagem. 2019;5(1):18.

12. Evans J, Papoulias C. Between funder requirements and 'jobbing scientists': the evolution of patient and public involvement in a mental health biomedical research centre - a qualitative study. Research Involvement and Engagement. 2020;6-12

13. Boylan A-M, Locock L, Staniszewska S. "About sixty per cent I want to do it": Health researchers' attitudes to, and experiences of, patient and public involvement (PPI) – A qualitative study.

14. INVOLVE survey of priorities for public involvement across the NIHR September 2011

15. Concordat for Engaging the Public with Research

16. Thompson J, Barber R, Ward PR, Boote JD, Cooper CL, Armitage CJ, et al. Health researchers' attitudes towards public involvement in health research. Health Expect 12(2):209-20 (2009).

17. McPin Foundation – Kirsten Morgan. Talking Point Papers 2: Patient and Public Involvement in Research- why not? 2016 https://mcpin.org/wp-content/uploads/talking-point-paper-2.pdf

18. McKenzie, A., Alpers, K., Heyworth, J. *et al.* Consumer and community involvement in health and medical research: evaluation by online survey of Australian training workshops for researchers. *Res Involv Engagem* 2016 **2,** 16

For peer review only

For peer review only - http://bmjopen.bmj.com/site/about/guidelines.xhtml

19. Dudley, L., Gamble, C., Allam, A. *et al.* A little more conversation please? Qualitative study of researchers' and patients' interview accounts of training for patient and public involvement in clinical trials. *Trials* **16,** 190 (2015) doi:10.1186/s13063-015-0667-4

20. Staniszewska S, Brett J, Simera I, Seers K, Mockford C, Goodlad S, Altman DG, Moher D, Barber R, Denegri S, Entwistle A. GRIPP2 reporting checklists: tools to improve reporting of patient and public involvement in research. BMJ. 2017 Aug 2;358.

21. Brett J, Staniszewska S, Simera I, Seers K, Mockford C, Goodlad S, Altman D, Moher D, Barber R, Denegri S, Entwistle AR. Reaching consensus on reporting patient and public involvement (PPI) in research: methods and lessons learned from the development of reporting guidelines. BMJ open. 2017 Oct 1;7(10):e016948.

22. NIHR. Experimental medicine. https://www.nihr.ac.uk/explore-nihr/support/experimental-medicine.htm

23. Martin GP. 'Ordinary people only': knowledge, representativeness, and the publics of public participation in healthcare. Sociology of health & illness. 2008 Jan;30(1):35-54.

24. Beresford P. User involvement in research: exploring the challenges. NT Res. 2003;8(1):36–46.

25. Beresford P. Public participation in health and social care: Exploring the co-production of knowledge. Front Sociol. 2019;3:41.

26. Doria N, Condran B, Boulos L, Maillet DG, Dowling L, Levy A. Sharpening the focus: differentiating between focus groups for patient engagement vs. qualitative research. Research Involvement and Engagement. 2018 Dec;4(1):1-8.

27. Boote J, Telford R, Cooper C. Consumer involvement in health research: a review and research agenda. Health Policy 61:213-36 (2002).

28. Petit-Zeman S. What do researchers really think about PPI? 2016. NIHR Oxford Biomedical Research Centre (2016) https://oxfordbrc.nihr.ac.uk/blog/what-do-researchers-really-think-about-ppi/

29. Crutch S, Herron D, Pickett J, Rosser S, Rossor M. Inspired by chance: valuing patients' informal contributions to research. BMJ. 2020 Dec 14;371.

30. In2Science. In2Science UK Impact Report 2020. https://in2scienceuk.org/wp-content/uploads/2020/11/In2scienceUK-Impact-Report-2020-digital.pdf

For peer review only

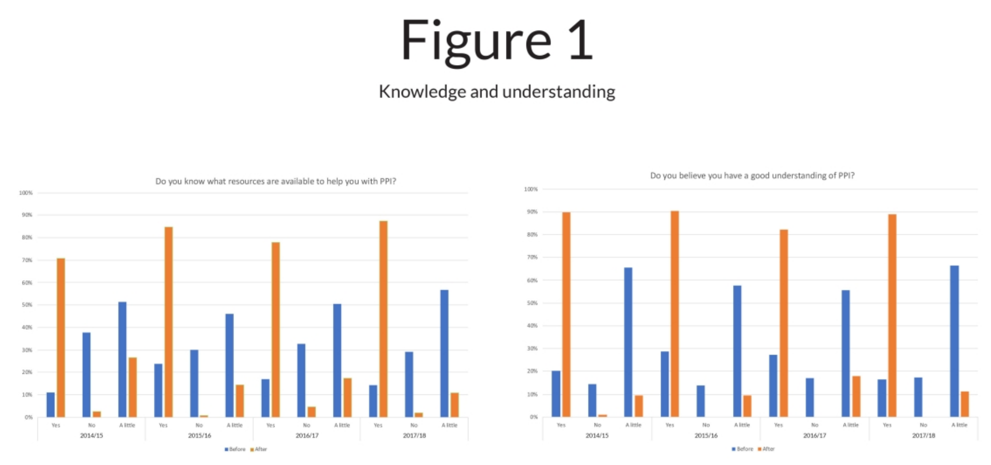

Figure 1: understanding and knowledge

296x142mm (300 x 300 DPI)

Figure 2

PPI skills - what researchers feel able to do before and after training (surveys 1, 2 and 3)

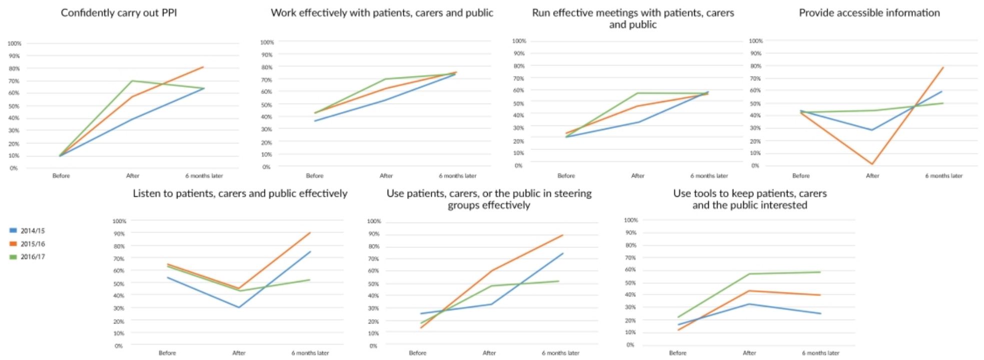

Figure 2: PPI skills - what researchers feel able to do before and after training (surveys 1,2 and3)

297x147mm (300 x 300 DPI)

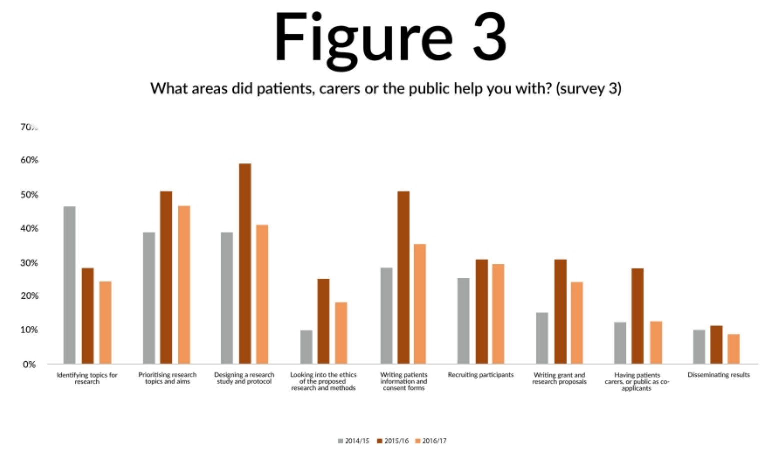

Figure 3: What areas did patients, carers or the public help you with (survey 3)

297x147mm (300 x 300 DPI)

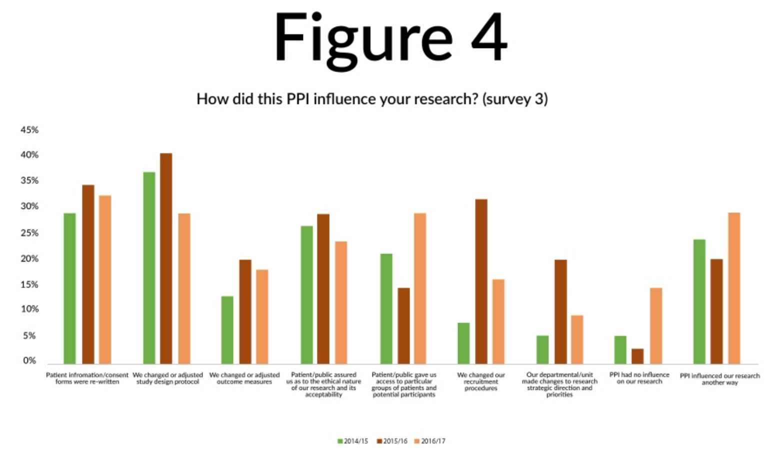

Figure 4: How did this PPI influence your research (survey3)

297x147mm (300 x 300 DPI)

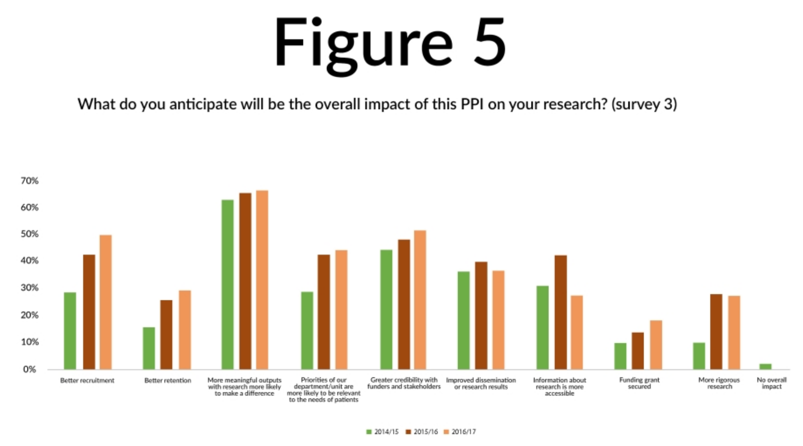

Figure 5: What do you anticipate will be the overall impact of this PPI on your research? (survey 3)

297x147mm (300 x 300 DPI)

Survey measures

Survey 1 (pre workshop)

1) Are you currently a Principal/Chief Investigator?

Yes

No

2) What is your professional role (tick one)?

Medical doctor/consultant

Nurse

Scientist

Administrator/study manager

Statistician/data manager

Graduate student

Other (please specify)

3) How would you describe your attitude towards Patient and Public Involvement (PPI)?

PPI enthusiast

Interested in PPI but have not yet done it

Indifferent

Negative

4) Do you feel able to: (please tick one or more)

Confidently carry out PPI

Work effectively with patients, carers and public$_{2}$

Run effective meetings with patients, carers and public

Listen to patients, carers and public effectively

Use patients, carers, or the public in steering groups effectively

Use tools to keep patients, carers and public interested

Provide accessible information

None of the above

Other – please explain:

5) Do you believe you have a good understanding of what is good practice in PPI?

Yes

No

A little

For peer review only

6) Do you know what resources are available to help you with PPI?

Yes
No
A little

7) Have you already actively involved patients, carers, or the public in your research?

Yes – go to a)
No

a) What areas did patients, carers, or the public help you with? (please tick one or more)

Identifying topics for research
Prioritising research topics and aims
Designing a research study and protocol
Looking into the ethics of the proposed research and methods
Writing patient information and consent forms
Recruiting participants
Writing grant and research proposals
Having patients, carers, or public as co-applicants
Disseminating results
Other - please explain:

Survey 2 (immediately after workshop)

1) How did you find the workshop? (Please mark the appropriate box for each row)

	Excellent	Good	Satisfactory	Poor
a. Overall workshop	☐	☐	☐	☐
b. Handouts	☐	☐	☐	☐
c. Delivery and presentation of workshop	☐	☐	☐	☐
d. Meeting room and facilities	☐	☐	☐	☐
e. Administration of workshop	☐	☐	☐	☐

For peer review only

2) How would you describe your attitude towards Patient and Public Involvement (PPI) now you have been to this workshop?

PPI enthusiast
Interested in PPI but have not yet done it
Indifferent
Negative

a) Has the workshop changed your attitude? (If yes, please explain)
Yes
No

3) Do you plan to involve patients, carers and the public in your research in the future?

Yes - go to a) and b)
No – go to c)

a) Will this workshop help you to action your plans?
Yes
No

b) How do you think this workshop will help you? (tick one or more)

I have more of an idea about how to involve patients, carers and public
It has given me more confidence to work with patients, carers and public
It has given me practical tips and ideas
I feel better equipped to work with patients, carers and public$_4$
Other – please explain:

4) What abilities and skills did you learn from our workshops? (please tick one or more)

Having confidence to carry out PPI
Understanding how to work with patients, carers and public
Running effective meetings with patients, carers and public
Listening to patients, carers and public more effectively
Using patients, carers and public in steering groups more effectively
Gaining tools to keep patients, carers and public interested
Providing accessible information
None of the above
Other – please explain

5) Would you recommend this workshop to other researchers?
Yes
No

For peer review only

6) Now that you have attended the workshop

a) Do you believe you have a good understanding of what is good practice in PPI?
Yes
No
A little

b) Do you know what resources are available to help you with PPI?
Yes
No
A little

For peer review only

7) Are you currently a Principal/Chief Investigator
Yes
No

8) What is your professional role (tick one)?

Medical doctor/consultant
Nurse
Scientist
Administrator/study manager
Statistician/data manager
Graduate student
Other (please specify): ___________________

9) Do you have any other comments or suggestions?

Survey 3 (6 months after workshop)

1. How would you describe your attitude towards Patient and Public Involvement (PPI) since attending the workshop? (Tick one box)

PPI enthusiast
Interested in PPI but have not yet done it
Indifferent
Negative

2. Since you attended the workshop in <month> have you actively involved patients or the public in your research? (Tick one box)

Yes – go to question 3 -
No, I haven't conducted any research since the workshop - go straight to question 7
No, PPI would not help my research - go straight to question 7
No, I don't feel able to actively involve the public - go straight to question 7
No, I didn't for other reasons - please specify and then go to question 7

3. What areas did patients, carers or the public help you with? (Tick relevant boxes)

Identifying topics for research
Prioritising research topics and aims
Designing a research study and protocol
Looking into the ethics of the proposed research and methods
Writing patient information and consent forms
Recruiting participants
Writing grant and research proposals
Having patients, carers, or public as co-applicants
Disseminating results
Other (please specify)

4. **Do you feel the workshop you attended in < month> helped you to involve patients, carers and the public in this way?**

Yes
No

5. How did this PPI influence your research? (Tick relevant boxes)

Patient information/consent forms were re-written
We changed or adjusted study design protocol
We changed or adjusted outcome measures
Patients/public assured us as to the ethical nature of our research and its acceptability
Patients/public gave us access to particular groups of patients and potential participants
We changed our proposed recruitment procedures
Lay people helped recruit participants

For peer review only

Our department/unit made changes to research strategic direction and priorities
PPI had no influence on our research
PPI influenced our research in another way (please specify)

6. What do you anticipate will be the overall impact of this PPI will have on your research? (Tick relevant boxes)

Better recruitment
Better retention
More meaningful outputs with research results more likely to make a difference
Priorities of our department/unit are more likely to be relevant to the needs of patients
Greater credibility with funders and stakeholders
Improved dissemination of research results
Information about research is more accessible
Funding grant secured
More rigorous research
No overall impact
PPI had a negative impact on our research

For peer review only

7. Which aspects of PPI do you now feel able to do and which would you like more training in?

	Able to do this	I need more training
Confidently carry out PPI	☐	☐
Work effectively with patients, carers and public	☐	☐
Run effective meetings with	☐	☐

patients, carers and public		
Listen to patients, carers and public effectively	☐	☐
Use patients, carers, or the public in steering groups effectively	☐	☐
Use tools to keep patients, carers and public interested	☐	☐
Provide accessible information	☐	☐
None of the Above	☐	☐
Other	☐	☐

For peer review only

8. Would you be interested in undertaking further training in PPI? (Tick one box)

Yes
No

9. If you would be interested in more training, what kind of training would you like? (Tick relevant boxes)

More introductory sessions on PPI
More advanced training
More practical training
Training where I can look at and discuss my own PPI plans
Training conducted by patients or members of the public
Sessions where I can exchange ideas with other researchers
Other (please specify)

For peer review only

10. Would you recommend the PPI workshop you attended to other researchers?

Yes
No
